# Transcriptional Profiling of Endobronchial Ultrasound-Guided Lymph Node Samples Aids Diagnosis of Mediastinal Lymphadenopathy

**DOI:** 10.1378/chest.15-0647

**Published:** 2016-01-12

**Authors:** Gillian S. Tomlinson, Niclas Thomas, Benjamin M. Chain, Katharine Best, Nandi Simpson, Georgia Hardavella, James Brown, Angshu Bhowmik, Neal Navani, Samuel M. Janes, Robert F. Miller, Mahdad Noursadeghi

**Affiliations:** a Department of Infection and Immunity, University College London, London, England; b Lungs for Living Research Centre, University College London, London, England; c Research Department of Infection and Population Health, University College London, London, England; d Department of Respiratory Medicine, Homerton University Hospital, London, England; e Department of Thoracic Medicine, University College London Hospital, London, England

**Keywords:** cancer, machine learning, sarcoidosis, transcriptome, tuberculosis, AUC, area under the curve, EBUS-TBNA, endobronchial ultrasound-guided transbronchial needle aspiration, PCA, principal component analysis, ROC, receiver operating characteristic, SVM, support vector machine

## Abstract

**Background:**

Endobronchial ultrasound (EBUS)-guided biopsy is the mainstay for investigation of mediastinal lymphadenopathy for laboratory diagnosis of malignancy, sarcoidosis, or TB. However, improved methods for discriminating between TB and sarcoidosis and excluding malignancy are still needed. We sought to evaluate the role of genomewide transcriptional profiling to aid diagnostic processes in this setting.

**Methods:**

Mediastinal lymph node samples from 88 individuals were obtained by EBUS-guided aspiration for investigation of mediastinal lymphadenopathy and subjected to transcriptional profiling in addition to conventional laboratory assessments. Computational strategies were used to evaluate the potential for using the transcriptome to distinguish between diagnostic categories.

**Results:**

Molecular signatures associated with granulomas or neoplastic and metastatic processes were clearly discernible in granulomatous and malignant lymph node samples, respectively. Support vector machine (SVM) learning using differentially expressed genes showed excellent sensitivity and specificity profiles in receiver operating characteristic curve analysis with area under curve values > 0.9 for discriminating between granulomatous and nongranulomatous disease, TB and sarcoidosis, and between cancer and reactive lymphadenopathy. A two-step decision tree using SVM to distinguish granulomatous and nongranulomatous disease, then between TB and sarcoidosis in granulomatous cases, and between cancer and reactive lymphadenopathy in nongranulomatous cases, achieved > 90% specificity for each diagnosis and afforded greater sensitivity than existing tests to detect TB and cancer. In some diagnostically ambiguous cases, computational classification predicted granulomatous disease or cancer before pathologic abnormalities were evident.

**Conclusions:**

Machine learning analysis of transcriptional profiling in mediastinal lymphadenopathy may significantly improve the clinical utility of EBUS-guided biopsies.

Endobronchial ultrasound-guided transbronchial needle aspiration (EBUS-TBNA) has transformed diagnostic evaluation of mediastinal lymphadenopathy in respiratory medicine. This has revolutionized lung cancer staging, providing a median sensitivity of 89% for detection of malignant cells, and also affords high sensitivity for detection of granulomatous lymphadenitis in sarcoidosis.^1^, ^2^, ^3^ Two specific diagnostic limitations have emerged as research priorities to maximize the benefits from EBUS-TBNA: (1) to increase sensitivity for detection of malignancy and hence the negative predictive value, to reduce the need for more invasive surgical sampling; and (2) to increase the sensitivity for detection of active TB and improve discrimination between TB and sarcoidosis in granulomatous lymphadenitis. This distinction currently relies on assessment of demographic risk of TB or microbiological confirmation, which is unavailable in > 50% of cases.^4^, ^5^

Genomewide transcriptional profiling can identify molecular signatures in peripheral blood or tumor specimens that could be used to improve diagnosis and risk stratification of patients with infectious and inflammatory diseases or to guide targeted therapeutic strategies for individuals with cancer.^6^, ^7^, ^8^, ^9^, ^10^ Feasibility of transcriptional profiling to detect gene expression associated with molecular pathogenesis of lung cancer in a small number of tumor-infiltrated nodes has been reported.^11^, ^12^ Granulomatous lymphadenitis has never been assessed by this method, but three previous studies reported extensive overlap of peripheral blood transcriptional signatures associated with TB and sarcoidosis.^13^, ^14^, ^15^ Although computational machine learning techniques were successfully applied to peripheral blood gene signatures to discriminate between TB and sarcoidosis cases, confidence in this approach has been partly undermined because of minimal overlap between the discriminating genes identified in each study.^13^, ^14^, ^15^ Since the inflammatory processes in sarcoidosis are reputed to be compartmentalized,^16^ we speculated that transcriptional profiles at the site of disease may offer better discrimination. In the present study we tested the hypothesis that genomewide transcriptional profiling of EBUS-TBNA samples will improve diagnostic differentiation of TB and sarcoidosis and detection of cancer in patients undergoing staging investigations.

## Materials and Methods

### Ethics Statement

The study was approved by the North London Research Ethics Committee (10/H0724/72). Written informed consent was obtained from all participants.

### Study Design

Lymph node samples were obtained from adult patients undergoing EBUS-TBNA for investigation of mediastinal lymphadenopathy (see e-Appendix 1 for a description of conventional assessments). Cases were classified according to predefined diagnostic criteria given in e-Table 1.^17^

### Transcriptional Profiling by cDNA Microarray

Lymph node cores harvested into RNALater (Qiagen) and homogenized in Qiazol (Qiagen) were used to obtain total RNA using the RNEasy Micro kit (Qiagen). Samples were processed for Agilent microarrays, and data were normalized as previously described (e-Appendix 1).^18^ Principal component analysis (PCA) was performed using the prcomp function in R to compare global gene expression profiles. Significant gene expression differences between samples were identified by *t* tests (*P* < .05) using MultiExperiment Viewer (version 4.6.0) and restricted to those with greater than twofold differences. Pathway overrepresentation analysis of differentially expressed genes was conducted using InnateDB, and transcriptional regulation of specific gene expression profiles was assessed by oPossum single transcription factor binding site enrichment analysis as previously described.^19^, ^20^ Additional details for the analysis of microarray data are provided in e-Appendix 1 and e-Figs 1, 2, 6. Microarray data are available in the ArrayExpress database (www.ebi.ac.uk/arrayexpress) under accession number E-MTAB-2547.

### Case Classification Using Machine Learning

Support vector machines (SVMs) for binary computational classification of high-dimensional data were trained to classify samples using selected gene signatures.^21^ Given a set of training data, an SVM establishes a model that optimizes separation of data points from two groups. The SVM algorithms were implemented using the kernlab package in R3.0.2 with a linear kernel, which allows the influence of each gene in the model to be weighted (see e-Appendix 1 for a detailed description of SVM classification). Bootstrap sampling with replacement was used to select training cases to optimize the SVM model. This model was then used to classify remaining data that were not included in the training cohort, and the process was repeated 100 times to evaluate its performance. Sensitivity and specificity values for multiple SVM models were then presented in receiver operating characteristic (ROC) curve analysis using the “pROC” package in R3.0.2. In additional assessments of the SVM models, we used leave-one-out cross-validation, in which the training set comprises all but one of the samples, which is then tested, and the process is repeated to test each individual sample. The minimum number of genes needed for accurate SVM classification was assessed by training the SVM on 10 randomly chosen cases to determine weight values for each gene, iteratively refined in 100 training sets. A cumulative sequence of genes ranked by weight was then used to determine SVM classification accuracy in distinct test cases using ROC curves.

## Results

### Transcriptomes Reflect Associated Molecular Pathologies

**Figure 1:**
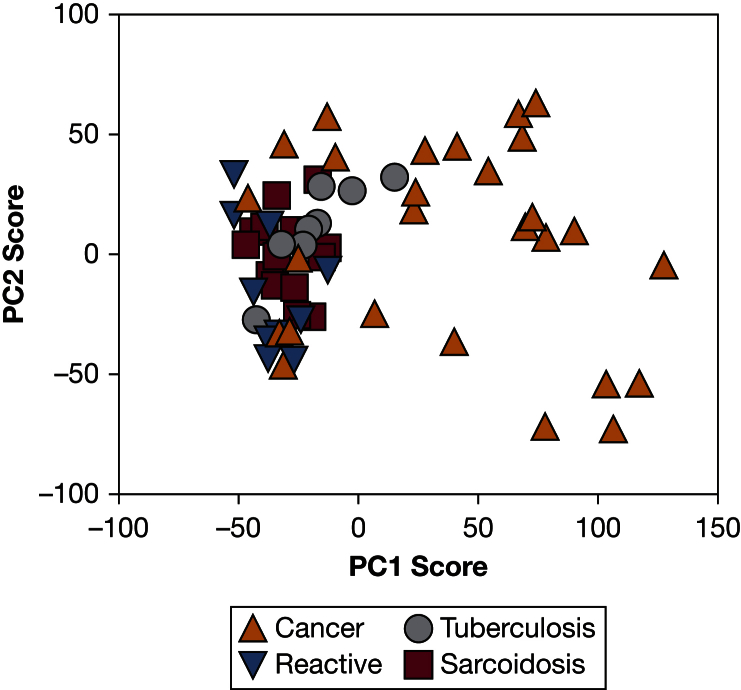
Clustering analysis of genomewide lymph node profiles does not distinguish disease groups. Comparison of genomewide transcriptional profiles of lymph node samples by principal component analysis (PCA) shows that the majority of cancer samples cluster away from all other disease groups in PC1, responsible for the greatest differences within the data. However, sarcoidosis, TB, and reactive lymph nodes as well as some cancer samples clustered together in both PC1 and PC2 in this analysis, which was unable to segregate individual disease groups. Each symbol represents a sample.

We performed transcriptional profiling of EBUS-TBNA samples from 88 patients (Table 1). Detailed information about study subjects is given in e-Table 2. First, we focused our analysis on patients with “definite” diagnoses of sarcoidosis, TB, cancer, or reactive lymphadenopathy, ascertained by routine clinical and laboratory assessments. Comparison of EBUS-TBNA genomewide data from these patients by PCA to visualize the greatest co-correlated differences between individual samples shows that sarcoidosis, TB, and reactive lymph node samples clustered together, and most but not all cancer samples clustered separately (Fig 1).

Despite the overlap in the clustering analysis described above, direct comparison of gene expression data identified differentially expressed genes between granulomatous and nongranulomatous lymph node samples (488 genes), between TB and sarcoidosis samples (58 genes), and between malignant and reactive samples (1,223 genes) (see e-Tables 3-5 for differentially expressed gene lists). Granulomatous lymph nodes were significantly enriched for genes associated with immunologic processes integral to cell-mediated immunity and granuloma formation and regulated by canonical transcription factors involved in proinflammatory and cytokine responses (e-Fig 3, e-Table 6). In keeping with previous peripheral blood transcriptional profiling studies,^13^, ^14^, ^15^ genomewide transcriptional profiles of TB and sarcoidosis lymph node samples were very similar. We identified only 16 genes with significantly higher expression in sarcoidosis and 42 genes with higher expression levels in TB lymph node samples (e-Fig 4).

In the nongranulomatous samples, malignant lymph node samples were significantly enriched for genes involved in cell cycle control and extracellular matrix interactions, consistent with processes related to cancer development and metastasis (e-Fig 5), and under the transcriptional regulation of Kruppel-like factors and other zinc finger protein family members (e-Table 7). These control cell proliferation, differentiation, migration, and pluripotency in normal tissues and regulate cancer cell proliferation, apoptosis, and metastasis in many human tumors.^22^

### Machine Learning Discriminates Diagnostic Categories

**Figure 2:**
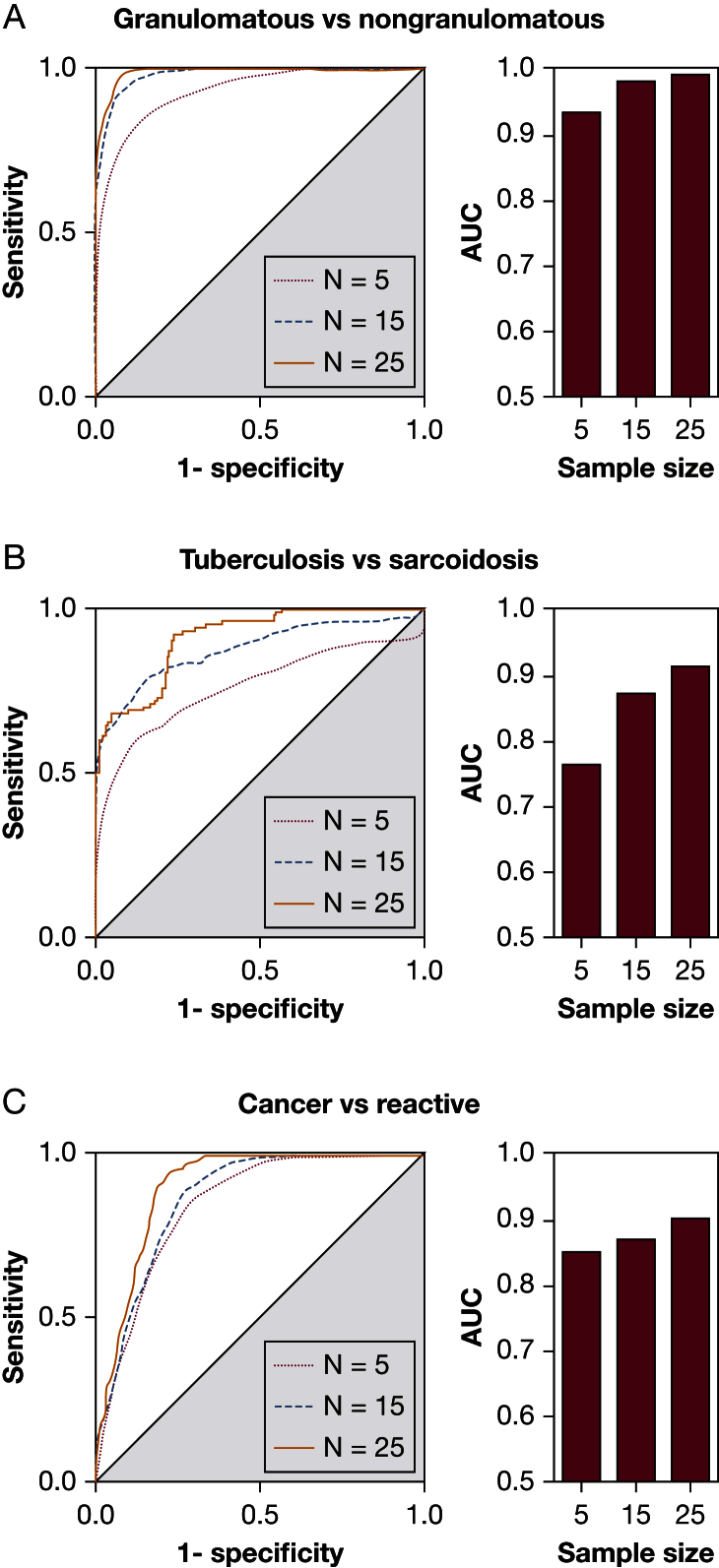
Support vector machines (SVMs) classification performance improves as cohort sample size increases. The performance of SVM to classify cases by training on signatures comprising differentially expressed genes (greater than twofold difference and *P* < .05, t test) between comparator groups using bootstrap sampling of five, 15, or 25 cases with replacement (100 iterations), is represented by receiver operating characteristic (ROC) curves. Increasing the training dataset sample size progressively improves the ability of SVMs to correctly distinguish granulomatous (n = 28) from nongranulomatous (n = 37) disease (A), sarcoidosis (n = 19) from TB (n = 9) (B), and malignant (n = 27) from reactive (n = 10) lymph nodes (C), with AUC values of > 0.9 once the cohort comprises 25 samples. AUC = area under the curve.

SVM are data-driven computational algorithms that can “learn” to discriminate between high-dimensional data such as genomewide transcriptomes. Therefore, we assessed the performance of SVM discrimination between diagnostic categories using the differentially expressed transcriptional signatures described above. In this analysis, we used repeated bootstrap subsampling cross-validation^23^, ^24^ with five, 15, or 25 training cases to evaluate the classification performance of the SVM, ensuring the test set was always independent of the training set. ROC curves showed that the SVM performance improved as the cohort sample size increased, achieving area under the curve (AUC) values > 0.9 in each case (Fig 2). A recognized limitation of SVM is risk of overfitting of training data, but the high levels of accuracy consistently achieved in multiple iterations using different combinations of training and test data strongly suggest overfitting does not confound the present analysis.

**Figure 3:**
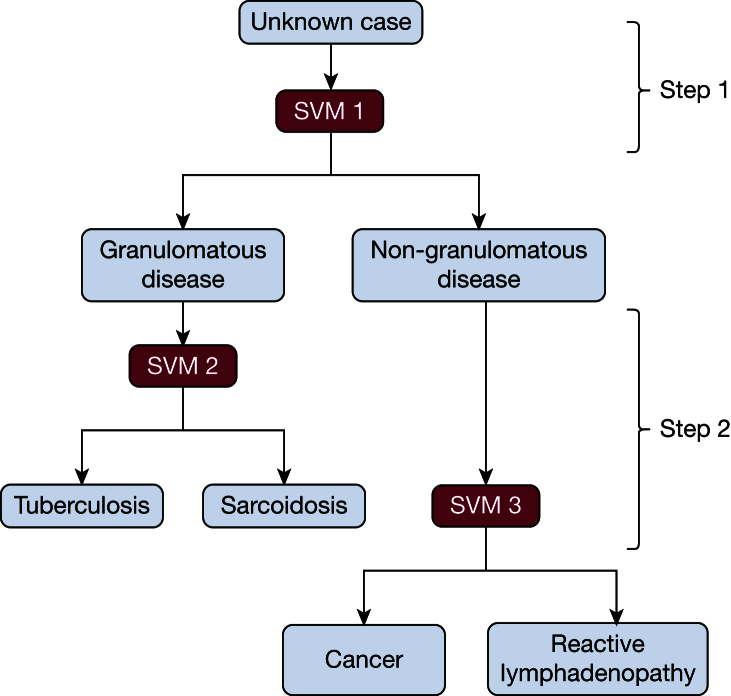
Support vector machines classification sequence. In this analysis, SVMs are trained using leave-one-out cross-validation. Step 1: Cases are subjected to initial SVM analysis using the 488-gene signature, which distinguishes granulomatous from nongranulomatous disease. Step 2: Samples classified as granulomatous disease subsequently undergo SVM testing using the 58-gene signature, which discriminates sarcoidosis from TB, and those classified as nongranulomatous disease undergo further SVM evaluation using the 1,223-gene signature, which distinguishes cancer from reactive lymph node. See Figure 2 legend for expansion of abbreviation.

Next we assessed the performance of SVM classification in a two-step decision tree sequence, using leave-one-out cross-validation as an alternative cross-validation strategy that optimizes the size of the training cohort (Fig 3). In step one, we classified each sample as granulomatous or nongranulomatous. In step two, samples classified as granulomatous were then subclassified as TB or sarcoidosis, and those classified as nongranulomatous were subclassified as cancer or reactive lymphadenopathy. We achieved excellent specificity across all four diagnostic groups and high sensitivity for detection of malignancy (93%) and sarcoidosis (85%) (Table 2, e-Tables 8, 9). This analysis was less sensitive for identification of reactive lymphadenopathy (80%) and TB (67%), but we noted a positive relationship between test sensitivity and sample size for each diagnosis (Table 2).

**Figure 4:**
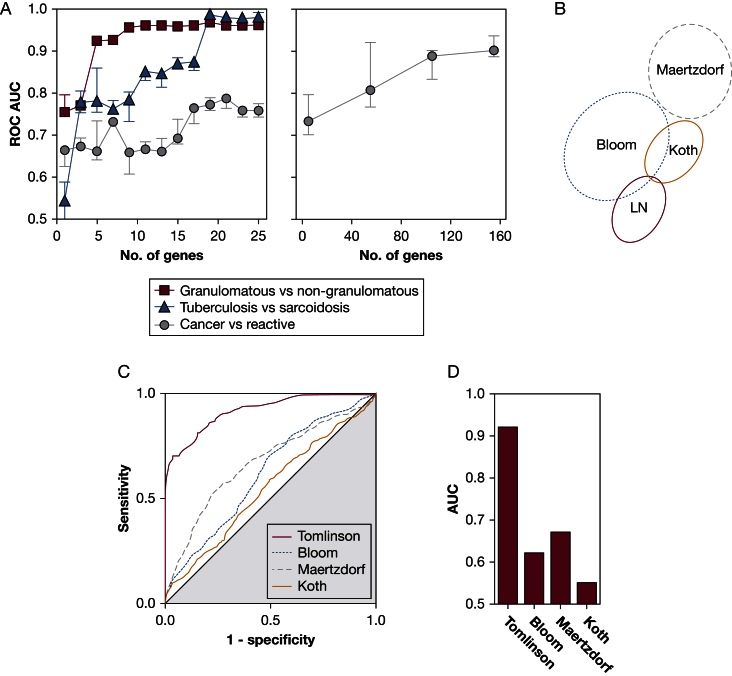
Lymph node transcriptional signatures show greater power to discriminate TB from sarcoidosis than peripheral blood signatures. A, Performance of cumulative genes in rank order of their weighting in each SVM is represented by the mean (± 95% CI) of ROC curve AUCs in 100 bootstrap subsampling cross-validation iterations. B, The published peripheral blood signatures (Maertzdorf, Bloom, Koth) and the LN signature identified in this study that differentiate TB from sarcoidosis exhibit minimal overlap. C, ROC curves represent the ability of SVM algorithms to classify sarcoidosis and TB lymph node samples when trained by bootstrap sampling with replacement on transcriptional signatures that distinguish sarcoidosis from TB lymph nodes (Tomlinson) or peripheral blood profiles (Bloom, Maertzdorf, Koth). D, The LN-derived gene signature performs significantly better (AUC = 0.92) than peripheral blood-derived signatures (AUC all < 0.7) in classifying TB and sarcoidosis samples. LN = lymph node. See Figure 2 legend for expansion of other abbreviations.

We also sought to identify the minimum number of genes needed to accurately classify cases using each SVM. The most discriminating genes were identified by their weighting in the training data sets using repeated bootstrap subsampling cross-validation (see e-Table 10 for lists of the most discriminating genes). Cumulative inclusion of these genes in order of their weighting in the SVM training data improved the ROC curve AUCs (Fig 4A). To achieve ROC curve AUC > 0.9, expression data from at least five genes were required to discriminate granulomatous and nongranulomatous cases, 19 genes to discriminate TB and sarcoidosis, and 150 genes to discriminate cancer and reactive cases.

Peripheral blood gene signatures that distinguish TB and sarcoidosis have been reported previously.^13^, ^14^, ^15^ These show only modest overlap with our lymph node-derived differential gene expression signature (Fig 4B) and clearly perform less well in discriminating TB from sarcoidosis lymph node samples by SVM classification (Figs 4C, 4D). This suggests that the gene signatures exhibit context specificity and that peripheral blood data may not faithfully reflect the site of disease.

### SVM Classification of Undiagnosed Lymphadenopathy

Finally, we tested the two-step SVM decision-tree sequence described above on cases in which a “definite” diagnosis could not be made at the time of EBUS-TBNA. We compared SVM classification of these samples with the final diagnosis based on follow-up data (Table 3). SVM analysis identified “granulomatous disease” in almost all specimens with histologic evidence of granulomas and two samples without granulomas on histology (Possible S2 and S3). A definite diagnosis of sarcoidosis was later confirmed in one case (Possible S2), after assessment of further lymph node samples obtained by mediastinoscopy revealed noncaseating granulomas. Another case (Probable TB2) was classified by SVM as “sarcoidosis” but subsequently confirmed as “TB.” The majority of “possible cancer” samples were classified as “reactive,” consistent with histologic assessments. However, SVM classified two cases as “cancer,” in the absence of histologic evidence. Subsequent examination of surgically resected specimens confirmed metastatic carcinoma in the lymph node, which had shown no evidence of malignancy on EBUS-TBNA 6 weeks previously (Possible C10). Strikingly, SVM also predicted the presence of “cancer” 4 months before tumor involvement was discernible on histology in another case (Possible C11). SVM predicted “cancer” in one further individual (U2) with a presumptive diagnosis of sarcoidosis and no evidence of malignancy during long-term follow up.

## Discussion

The development of EBUS has revolutionized investigation of mediastinal lymphadenopathy by facilitating minimally invasive sampling of lymph nodes at the site of disease. Transcriptional profiling of EBUS-TBNA in granulomatous and malignant lymphadenopathy revealed clear evidence of the key biologic processes relevant to each disease. Samples from granulomatous diseases were enriched for immune cell recruitment and activation as well as antigen presentation and interferon-γ signaling, which characterize granulomatous inflammation.^25^ Malignant lymph node samples were enriched for molecules involved in control of cell division and interactions with the extracellular matrix or adjacent cells, the molecular mechanisms that underpin cancer development and metastasis. Bioinformatic analysis of the transcriptional control of genes involved in these processes confirmed the importance of nuclear factor-κB as a principal regulator of inflammatory responses in granulomatous disease and Kruppel-like factors in transcriptional regulation of key events in tumor generation in humans in vivo.^22^ Macroscopic blood contamination of EBUS-TBNA was variable, but globin transcript levels were comparable between the diagnostic groups and therefore did not produce a systematic bias. Although globin depletion is advocated in blood samples to improve sensitivity,^26^ biologically plausible gene expression differences between groups in our study were still evident. Nonetheless, assessment of globin depletion or RNA sequencing to increase sensitivity should be considered in future studies.

The similarity between TB and sarcoidosis profiles suggests that by the time of clinical presentation, the molecular pathology in these diseases is comparable. Sarcoidosis samples showed higher expression of genes with putative roles in granuloma formation, including cathepsin K, transmembrane 7 superfamily member 4, and chemokine (C-C motif) ligand 21.^27^, ^28^, ^29^ We hypothesize that this may reflect the more organized, noncaseating granuloma phenotype typical of sarcoidosis.^30^ The most highly expressed gene in sarcoidosis compared with TB lymph nodes was chitotriosidase (CHIT)1, which encodes a lysosomal hydrolase that degrades fungal cell wall chitin.^31^ Elevated levels of chitotriosidase have also been reported in BAL fluid and serum of individuals with sarcoidosis.^32^, ^33^ A putative role for inhaled antigens is recognized in sarcoidosis.^34^ Therefore, aberrant immune responses to fungi in pathogenesis of sarcoidosis merit further investigation.

The performance of SVM using differential gene expression signatures to distinguish between granulomatous and nongranulomatous disease, cancer and reactive lymphadenopathy, and TB and sarcoidosis showed excellent promise to improve diagnostic classification, providing AUC statistics of > 0.9 in each case. Importantly, the SVM models identified the most influential genes and opportunity to test the feasibility of using targeted transcriptional analysis rather than genomewide technologies in future studies. Our analysis suggested that this might be possible for discriminating granulomatous from nongranulomatous disease and TB from sarcoidosis using multiplex quantitative polymerase chain reaction analysis of 20 to 30 genes. However, expression data from more than 150 genes were required to discriminate cancer and reactive lymphadenopathy.

The application of SVM in a two-step decision tree model was significantly more sensitive than mycobacterial culture for identification of TB and equivalent to histologic detection of noncaseating granulomas for sarcoidosis.^4^, ^5^, ^35^, ^36^ Notably, SVM predicted the presence of granulomatous disease in one EBUS-guided biopsy with no granulomas on histology, from an individual subsequently diagnosed with sarcoidosis after lymph node samples obtained by mediastinoscopy confirmed granulomatous inflammation. Importantly, differentially expressed genes that distinguished sarcoidosis from TB lymph node transcriptional profiles performed better than previously published gene signatures derived from peripheral blood,^13^, ^14^, ^15^ suggesting that peripheral blood does not wholly reflect the profiles at the site of disease and strengthening the case for lymph node sampling for maximum diagnostic accuracy.

SVM also classified specimens from two individuals undergoing EBUS-guided lymph node sampling for lung cancer staging as “cancer,” despite no histologic evidence of malignancy. In keeping with this, SVM classification was slightly more sensitive than the reported median sensitivity of histologic identification of malignancy.^2^ In both cases, further biopsies taken from these nodes 6 weeks to 4 months after the initial specimens, demonstrated tumor infiltration that may reflect progression of micrometastatic disease. These examples highlight the potential power of combining transcriptional profiling with a computational classification algorithm to provide a more sensitive approach for identification of subtle molecular indicators of granulomatous disease or malignancy that are discernible before histologic or cytologic abnormalities become evident. Importantly, this strategy has the potential to identify individuals who might benefit from more frequent surveillance and to improve the current low negative predictive value of 40% for EBUS-guided lymph node sampling in isolated mediastinal lymphadenopathy,^37^ which will avoid unnecessary invasive mediastinoscopy procedures and minimize delays in definitive treatments. We recognize that SVM analysis of transcriptome data did not correctly classify every case in our study; therefore, this approach does not currently supersede clinical assessment and laboratory investigations. However, both accuracy and sensitivity of SVM classification improved as the training dataset sample size increased, suggesting the potential for more precise discrimination and increased sensitivity with expansion of the current cohort. Our data pave the way for larger-scale observational cohorts to validate the findings presented here and controlled trials to investigate the impact of this approach on clinical outcomes.

## Conclusions

We propose that transcriptional profiling of lymph node samples from the site of disease combined with machine learning data analysis offers a novel strategy with molecular-level resolution that could be applied to augment conventional investigation of clinically ambiguous cases of mediastinal lymphadenopathy. This merits further evaluation in future large-scale clinical trials.

## Figures and Tables

**Table 1:** Summary of Study Subjects’ Demographic Data

Diagnosis	No.	Age Range, y	Sex M (F)	Ethnicity
Definite sarcoidosis	19	24-80	12 (7)	16 Eurasian, 3 African
Definite TB	9	21-71	8 (1)	7 Eurasian, 2 African
Reactive	10	33-78	9 (1)	10 Eurasian
Definite cancer	27	49-86	16 (11)	25 Eurasian, 1 East Asian, 1 African
Possible sarcoidosis	3	38-50	3 (0)	2 Eurasian, 1 Latin American
Probable TB	2	30-35	0 (2)	2 Eurasian
Possible cancer	12	56-82	5 (7)	12 Eurasian
Undetermined	6	44-58	2 (4)	5 Eurasian, 1 African

**Table 2:** Sensitivity and Specificity of SVM Classification

Diagnosis	No.	Sensitivity, %	Specificity, %
Sarcoidosis	19	85	96
TB	9	67	98
Reactive	10	80	93
Cancer	27	93	92

SVM = support vector machine.

**Table 3:** SVM Classification of Cases Without a Definite Clinical Diagnosis

Diagnosis	Clinical Question	Histology	SVM 1	SVM 2	SVM 3	Clinical Outcome
Possible S1	Sarcoidosis?	G	NG	…	R	Clinical diagnosis of sarcoidosis. Spontaneous resolution of symptoms, under observation only.
Possible S2	Sarcoidosis?	NG	G	S	…	Definite sarcoidosis confirmed by noncaseating granulomas on lymph node sample from mediastinoscopy. Clinical improvement with steroid treatment.
Possible S3	Sarcoidosis?	NG	G	S	…	Spontaneous improvement in clinical symptoms and chest radiograph lymphadenopathy.
Probable TB1	TB?	G	G	TB	…	Good clinical and radiologic response to empirical TB treatment.
Probable TB2	TB?	G	G	S	…	Pleural fluid acid- alcohol-fast bacilli positive, *Mycobacterium tuberculosis* culture negative. Good clinical response to empirical TB treatment.
Possible C1	Metastatic endometrial cancer?	G	G	…	R	Palliative chemotherapy for presumed lung metastases.
Possible C2	Sarcoidosis/lymphoma?	G	G	…	R	Died of non-Hodgkin’s lymphoma diagnosed on bone marrow biopsy.
Possible C3	Metastatic bowel cancer?	G	G	…	R	Patient declined empirical TB treatment and did not attend further respiratory follow-up appointment.
Possible C4	Metastatic bowel cancer?	G	G	…	R	Died.
Possible C5	Metastatic lung cancer?	NG	NG	…	R	Developed further lung lesion and fluorodeoxyglucose-avid lymph node following wedge resection of right lower lobe tumor.
Possible C6	Cancer?	NG	NG	…	R	Remains in remission from lung cancer—no further treatment.
Possible C7	Metastatic lung cancer?	NG	NG	…	R	Died.
Possible C8	Metastatic bladder/renal cancer?	NG	NG	…	R	Clinically well after neoadjuvant chemotherapy, right nephroureterectomy, cystoprostatectomy, and ileal conduit formation.
Possible C9	Metastatic lung cancer?	NG	NG	…	R	Neoadjuvant chemotherapy, left upper lobe resection, consolidation chemotherapy. Clinically stable but mediastinal lymphadenopathy still evident on CT scan.
Possible C10	Metastatic lung cancer?	NG	NG	…	C	Left upper lobe resection specimen showed metastatic carcinoma in the lymph node, which had shown no histologic evidence of malignancy on samples obtained via EBUS.
Possible C11	Metastatic lung cancer?	NG	NG	…	C	Previous right middle lobe resection for lung adenocarcinoma and had confirmed metastatic adenocarcinoma on a second EBUS procedure performed 4 mo later due to progressive lymph node enlargement.
Possible C12	Metastatic bladder cancer?	NG	NG	…	R	Clinically stable, static appearance of lymphadenopathy on interval CT scan; discharged from respiratory follow-up.
U1	Sarcoidosis/TB?	G	G	S	…	Empirical TB treatment stopped after 2 mo as no response. Now under observation—remains clinically stable.
U2	Sarcoidosis?	NG	NG	…	C	Presumptive diagnosis of sarcoidosis-induced peripheral neuropathy. Minor clinical improvement with steroid and cyclophosphamide treatment.
U3	Sarcoidosis/lymphoma?	NG	G	S	…	Unknown—patient did not attend follow-up clinical appointments.
U4	Sarcoidosis?	G	G	S	…	Remains clinically stable—under observation only.
U5	Sarcoidosis/TB?	G	G	S	…	Spontaneous improvement in clinical symptoms and chest radiograph lymphadenopathy. Subsequently completed 6 mo empirical TB treatment (Mantoux 45 mm, interferon-γ release assay negative). Now well—discharged.
U6	Sarcoidosis/TB/lymphoma?	NG	NG	…	R	Clinically well after 6 mo empirical TB treatment—discharged.

C = cancer; EBUS = endobronchial ultrasound; G = granulomatous; NG = nongranulomatous; R = reactive; S = sarcoidosis; U = undetermined, See Table 2 legend for expansion of other abbreviation.

## Supplementary Data

e-Online Data
