# Supplementary material for: Transcriptional Profiling of Endobronchial Ultrasound-Guided Lymph Node Samples Aids Diagnosis of Mediastinal Lymphadenopathy
Source: Chest. 2016 Jan 12;149(2):535–44. doi: 10.1378/chest.15-0647 (PMC4740456; doi:10.1378/chest.15-0647)
Supplement: e-Online Data [file mmc1.pdf]

CHEST Online Supplement

Transcriptional Profiling of Endobronchial Ultrasound-Guided Lymph Node Samples Aids Diagnosis of Mediastinal Lymphadenopathy

Gillian S. Tomlinson, PhD; Niclas Thomas, PhD; Benjamin M. Chain, PhD; Katharine Best, MRes; Nandi Simpson, PhD; Georgia Hardavella, PhD; James Brown, MD; Angshu Bhowmik, MD; Neal Navani, PhD; Samuel M. Janes, PhD; Robert F. Miller, MBBS; and Mahdad Noursadeghi, PhD

CHEST 2016; 149(2):535-544

Online supplements are not copyedited prior to posting and the author(s) take full responsibility for the accuracy of all data.

© 2016 AMERICAN COLLEGE OF CHEST PHYSICIANS. Reproduction of this article is prohibited without written permission from the American College of Chest Physicians. See online for more details. **DOI:** 10.1378/chest.15-0647

e-Appendix 1. Supplementary methods

Conventional investigation of mediastinal lymphadenopathy

Routine haematology and biochemistry blood investigations were performed in all individuals. Serum angiotensin converting enzyme levels were also evaluated in some definite and possible sarcoidosis cases. All study participants underwent thoracic computed tomography (CT) scanning to define mediastinal lymphadenopathy. Lymph node samples obtained by endobronchial ultrasound transbronchial aspiration (EBUS-TBNA) were subjected to bacterial culture, acid and alcohol fast bacilli (AAFB) staining, mycobacterial culture, cytological and where possible, histological examination. Immunohistochemistry staining was performed to verify cancer diagnoses in cases where histological evaluation was inconclusive and lung adenocarcinoma samples were screened for epidermal growth factor receptor (EGFR) mutations using the *therascreen* EGFR RGQ PCR Kit (Qiagen).

Whole genome transcriptional profiling

Lymph node cores were disrupted in QIAzol (Qiagen) using a homogeniser (Omni International). Total RNA was purified from these samples using the RNEasy Micro kit (Qiagen), according to the manufacturer's instructions. Samples were subjected to DNase treatment using the TURBO DNA-free kit (Ambion). RNA quantity and integrity were measured electrophoretically using the Agilent RNA 6000 Nano assay/Agilent 2100 Bioanalyzer. 25-100 ng total RNA was amplified, reverse transcribed into cDNA and then to cRNA and labelled with Cy5 or Cy3 using the Agilent Low RNA Input Linear Amplification Kit. Purification, labelling intensity and RNA concentration were verified using the NanoDrop ND-1000 UV-VIS Spectrophotometer (Thermo Scientific) in order to ensure that only samples with a specific activity ≥8 pmol Cy3 or Cy5 dye per microgram cRNA were used for microarray analysis. Equal concentrations of Cy5-labelled and Cy3-labelled cRNA samples were mixed and hybridized to Agilent 8x60k arrays according to the manufacturer's instructions. Array images were acquired with Agilent's dual-laser microarray scanner G2565BA and signal data were collected with Agilent Feature Extraction software (v10.7.1.1). Median Cy3 and Cy5 signal intensity was Log transformed and normalized using LOESS local linear regression against the mean signal of all the samples using the R package agilp (http://www.bioconductor.org/packages/release/bioc/html/agilp.html). Microarray data were subject to quality control testing before inclusion in the analyses presented here. Firstly, the customised Agilent Feature Extraction software quality control report provided an assessment of linearity of observed versus expected data obtained from "spike in" controls. Secondly, normalised data were subjected to Sum of Squared Error analysis using the AALoess script within agilp in order to identify outliers. This strategy identified one outlier sample which was excluded from further analysis. Probes without Refseq or gene symbol annotations and those which showed low expression values (relative expression <6.5) or whose

Online supplements are not copyedited prior to posting and the author(s) take full responsibility for the accuracy of all data.

expression varied by less than two fold across all samples (17,914 in total) were excluded from subsequent analyses.

Blood contamination of some EBUS-TBNA specimens was evident macroscopically. We did not subject these to globin depletion, which is advocated for whole blood transcriptomics. Instead, we excluded the possibility of any systematic confounding of our analysis by blood contamination, by showing that expression of haemoglobin encoding genes in all transcriptional data used in the analysis of definite case was equivalent across all four diagnostic groups (e-Figure 1). We also excluded the possibility that gene expression differences were due to batch effects by performing principal component analysis (PCA) of genome-wide transcriptomic data which showed no evidence of clustering by individual batches of transcriptional arrays (e-Figure 2). We did notice differences in the gender distribution of sarcoidosis cases (63% male) compared to TB cases (89% male) and reactive lymph node samples (90% male) compared to cancer samples (59% male). However, PCA of differentially expressed genes between TB and sarcoidosis and between cancer and reactive lymph nodes did not reveal any global confounding effects of gender (e-Figure 6).

Identification of the minimum number of genes for SVM classification

Gene subset selection was conducted to determine if a more parsimonious model containing fewer genes could be used to classify cases. Initially, an SVM model was trained on 10 training cases to determine the weight values corresponding to each gene. SVM models identify the optimal separating hyperplane that splits data points from the two classes in the training data. This hyperplane is defined by an equation $\sum_{i=1}^{i=n} a_i x_i = 0$ where a_i are weights assigned to each dimension of the data, in this case to each gene. The weights (which are determined by the SVM algorithm) can be considered as capturing the importance of each gene in determining classification. In order to obtain the weights, we used the *kernlab* package in R (http://cran.r-project.org/web/packages/kernlab/index.html). This can be implemented by calling the "ksvm" function: **mymodel <- ksvm(Class ~ ., data = my.data, type = "C-svc", kernel = "vanilladot")**, where my.data is the training data, and then: **weights <- colSums(coef(mymodel)[[1]] * data[SVindex(mymodel),-c(1)])** to calculate the weights.

Online supplements are not copyedited prior to posting and the author(s) take full responsibility for the accuracy of all data.

e-Figure 1

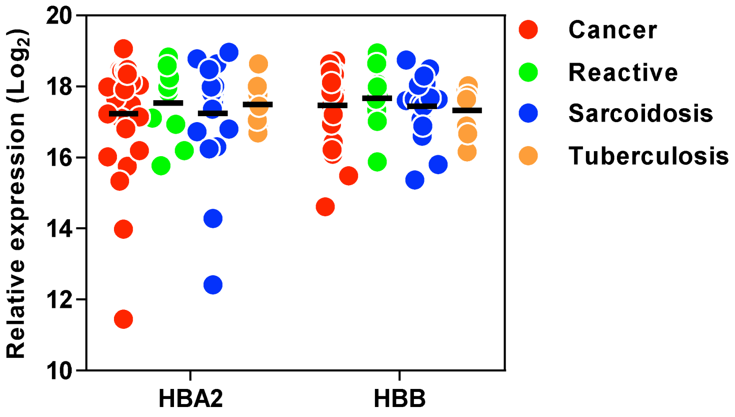

e-Figure 1. Equivalent haemoglobin gene expression in lymph node samples from all diagnostic groups.

Relative expression levels of haemoglobin encoding genes haemoglobin, alpha 2 (HBA2) and haemoglobin, beta (HBB) in lymph node specimens from definite cases (n=65) are shown. There were no significant differences in expression of either HBA2 ($p=0.9211$, one-way ANOVA) or HBB ($p=0.8740$, one-way ANOVA) between any of the groups. Each symbol represents an individual sample and bars represent the mean for each group.

e-Figure 2

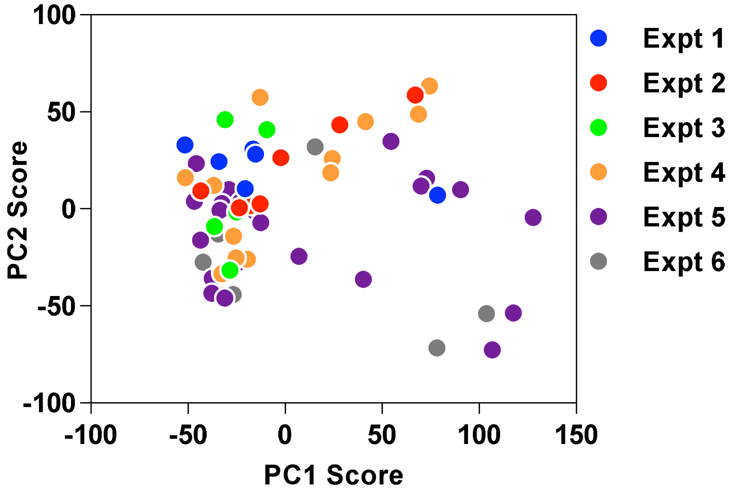

e-Figure 2. Clustering analysis of genome-wide lymph node profiles is not confounded by experimental batch effects.

Comparison of genome-wide transcriptional profiles of lymph node samples from definite cases (n=65) by principal component analysis shows no evidence of sample clustering by experiment. Each symbol represents a sample.

Online supplements are not copyedited prior to posting and the author(s) take full responsibility for the accuracy of all data.

e-Figure 3

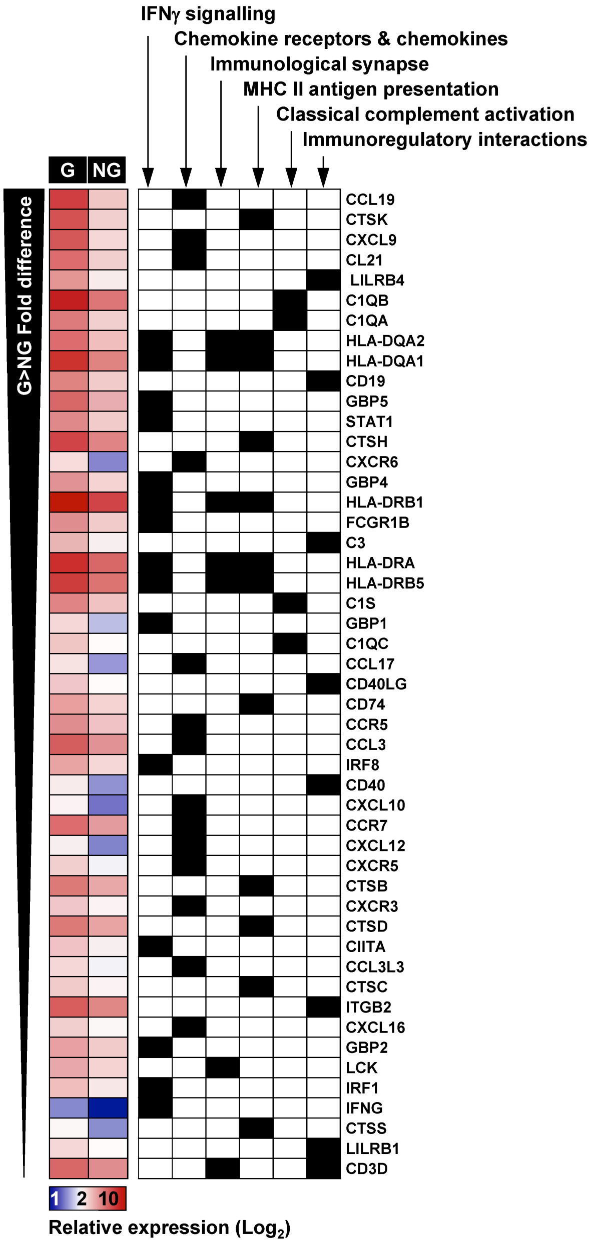

e-Figure 3. Granulomatous lymph node profiles are enriched for immunological processes involved in granuloma formation.

Mean relative expression levels are shown for granulomatous (n=28) and non-granulomatous (n=37) lymph node transcriptional profiles for the genes that were significantly more highly expressed (> two fold difference and $p<0.05$, t-test) and represented within the most highly enriched pathways in granulomatous compared to non-granulomatous lymph node samples.

e-Figure 4

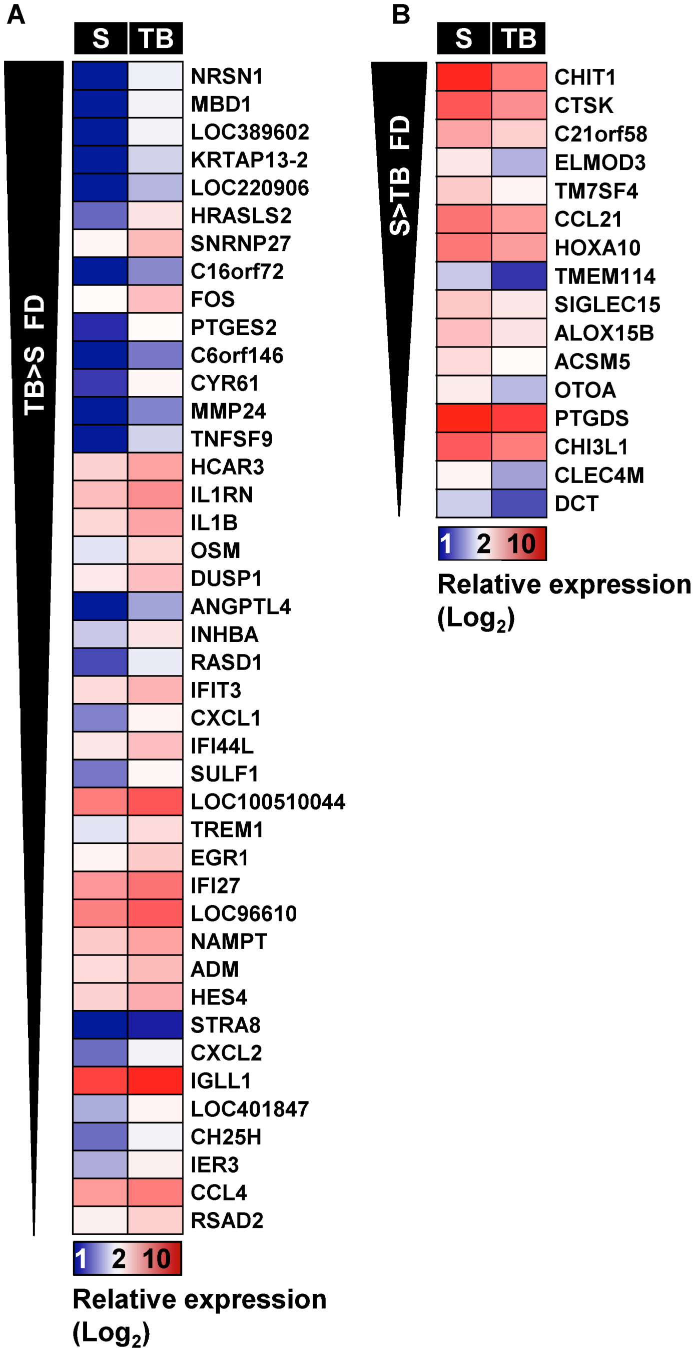

e-Figure 4. Gene expression differences between sarcoidosis and tuberculosis lymph nodes.
Heat maps show mean relative expression levels for sarcoidosis (n=19) and tuberculosis (n=9) lymph node profiles of (A) 42 genes that were significantly more highly expressed in tuberculosis samples and (B) 16 genes that showed significantly higher expression in sarcoidosis samples (>two fold difference and $p<0.05$, t-test).

Online supplements are not copyedited prior to posting and the author(s) take full responsibility for the accuracy of all data.

e-Figure 5

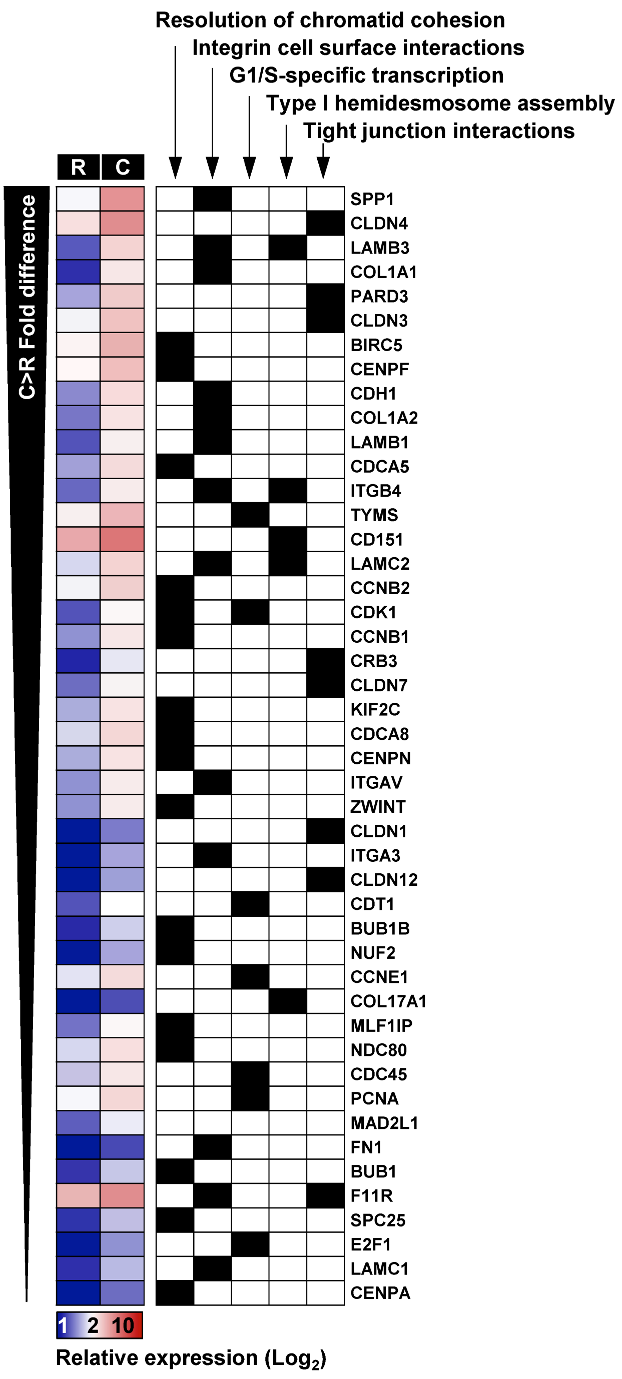

e-Figure 5. malignant lymph node profiles are enriched for molecular mechanisms of cancer development and metastasis

Mean relative expression levels are presented for malignant and reactive lymph node specimens for genes within the most highly enriched pathways that were expressed at significantly higher levels in malignant (n=27) compared to reactive (n=10) lymph nodes samples.

Online supplements are not copyedited prior to posting and the author(s) take full responsibility for the accuracy of all data.

e-Figure 6

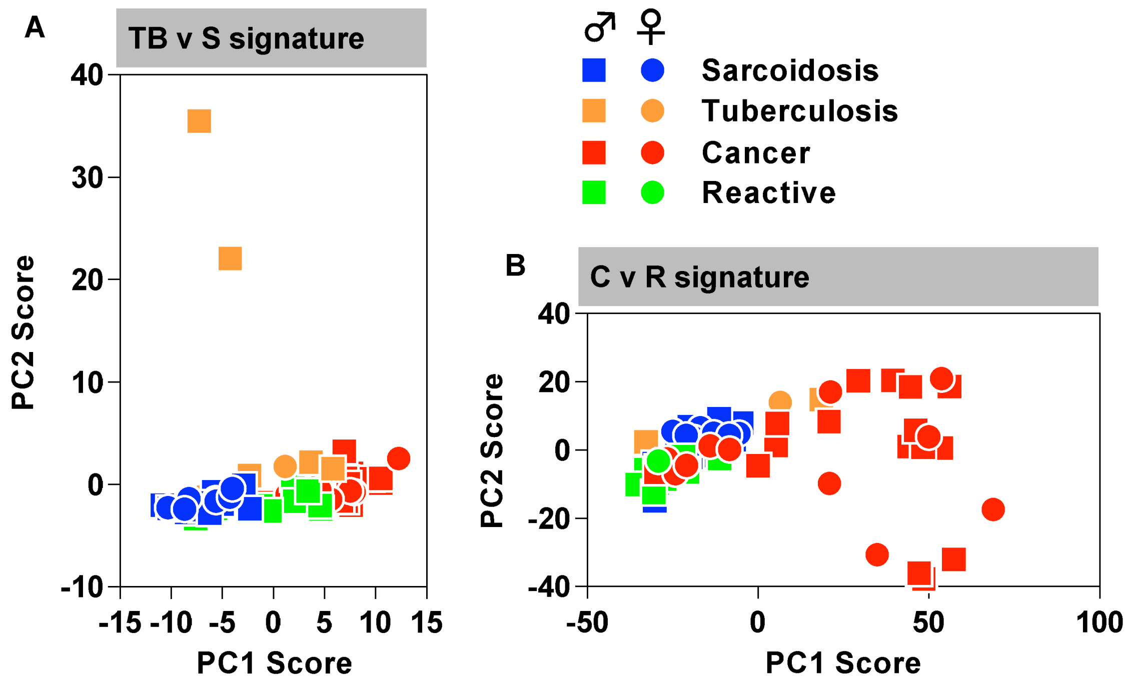

e-Figure 6. Gender does not confound lymph node transcriptomic analysis

Principal component analysis of lymph node transcriptional profiles from definite cases (n=65) specifically restricted to genes that show significantly different expression (>two fold difference and $p<0.05$, t test) between (A) sarcoidosis (n=19) and tuberculosis (n=9) or (B) malignant (n=27) and reactive nodes (n=10) shows no evidence of sample clustering by gender in any diagnostic group. However, this analysis does not reliably segregate disease groups. Each symbol represents a sample.

Online supplements are not copyedited prior to posting and the author(s) take full responsibility for the accuracy of all data.

e-Table 1. Case definitions.

Diagnosis	Definition
**Definite sarcoidosis**	Culture negative lymph node samples containing non-caseating granulomas, no evidence of malignancy on cytology or histology, with consistent clinical and radiological features of at least six months duration and no anti-tuberculosis therapy
**Possible sarcoidosis**	Consistent clinical and radiological features for at least six months, no evidence of granulomatous inflammation or malignancy on lymph node cytology or histology and no anti-tuberculosis therapy
**Definite tuberculosis**	Culture positive lymph node samples with clinical and radiological response to anti-tuberculosis chemotherapy
**Probable tuberculosis**	Culture negative lymph node samples with granulomatous inflammation on cytology or histology, and demographic risk for tuberculosis with clinical and radiological response to anti-tuberculosis chemotherapy
**Definite cancer**	Malignancy on lymph node cytology or histology
**Possible cancer**	Contemporaneously (within three months) confirmed diagnosis of malignancy on cytology or histology of tissue from another anatomical site, in the context of intra-thoracic lymph node enlargement or increased tracer uptake on positron emission tomography (PET) imaging
**Reactive**	Culture negative lymph node samples with no features of granulomatous inflammation or malignancy on cytology or histology and no evidence of malignancy, sarcoidosis or tuberculosis in any other anatomical site during six months follow up
**Undetermined**	Cases which did not fulfil the diagnostic criteria for inclusion in any of the above groups

Online supplements are not copyedited prior to posting and the author(s) take full responsibility for the accuracy of all data.

E-TABLE 2. STUDY PARTICIPANTS DEMOGRAPHIC AND CLINICAL DATA.

Study code	Age	Gender	Ethnicity	Clinical query	Loefgren's syndrome	Serum ACE	Other organ involvement	Histology	Smear result	Mtb culture result	Sensitivities	Reason for diagnostic ambiguity
**S1**	31	Male	Eurasian	Sarcoidosis?	Information unavailable	Information unavailable	Ocular	N-C granulomas	Negative	Negative	NA	NA
**S2**	58	Female	Eurasian	Sarcoidosis?	Yes	Information unavailable	Information unavailable	N-C granulomas, favours sarcoidosis	Negative	Negative	NA	NA
**S3**	43	Male	Eurasian	Sarcoidosis?	Information unavailable	Information unavailable	Information unavailable	Granulomatous lymphadenitis	Negative	Negative	NA	NA
**S4**	49	Male	African	Sarcoidosis?	No	63	Cardiac	N-C granulomas-favours sarcoidosis	Negative	Negative	NA	NA
**S5**	45	Male	Eurasian	Sarcoidosis?	No	Information unavailable	Ocular, cranial nerve	N-C granulomas, favours sarcoidosis	Negative	Negative	NA	NA
**S6**	31	Female	Eurasian	Sarcoidosis?	Information unavailable	Information unavailable	Information unavailable	N-C granulomatous inflammation	Negative	Negative	NA	NA
**S7**	60	Male	Eurasian	Sarcoidosis?	Yes	7	No	N-C granulomatous inflammation, favours sarcoidosis	Negative	Negative	NA	NA
**S8**	65	Male	Eurasian	Sarcoidosis?	No	Information unavailable	No	N-C granulomas	Negative	Negative	NA	NA
**S9**	54	Female	Eurasian	Sarcoidosis?	No	116	CNS, bone, abdominal lymph node	N-C granulomas, consistent with sarcoidosis	Negative	Negative	NA	NA
**S10**	24	Male	Eurasian	Sarcoidosis?	Yes	128	No	N-C granulomas & giant cells, favours sarcoidosis	Negative	Negative	NA	NA
**S11**	27	Female	African	Sarcoidosis?	Yes	117	Ocular, nasopharynx, submandibular and parotid gland	Epithelioid granulomas	Negative	Negative	NA	NA
**S12**	45	Female	African	Sarcoidosis /TB?	No	104	Ocular, cranial nerve, gastric	N-C granulomas, favours sarcoidosis	Negative	Negative	NA	NA
**S13**	76	Male	Eurasian	Sarcoidosis /TB/ lymphoma?	No	<4	Abdominal and inguinal lymph node	N-C granulomas	Negative	Negative	NA	NA
**S14**	24	Female	Eurasian	Sarcoidosis?	Information unavailable	Information unavailable	Information unavailable	Well-formed N-C granulomas	Negative	Negative	NA	NA
**S15**	41	Male	Eurasian	Sarcoidosis?	Yes	71	Information unavailable	N-C granulomas	Negative	Negative	NA	NA
**S16**	35	Male	Eurasian	Sarcoidosis?	Information unavailable	Information unavailable	Ocular	N-C granulomas	Negative	Negative	NA	NA
**S17**	80	Female	Eurasian	Sarcoidosis?	No	Information unavailable	No	N-C granulomas	Negative	Negative	NA	NA
**S18**	70	Male	Eurasian	Sarcoidosis?	No	47	No	N-C granulomas consistent with sarcoidosis	Negative	Negative	NA	NA
**S19**	31	Male	Eurasian	Sarcoidosis?	No	Information unavailable	Information unavailable	Well-formed N-C granulomas & MGC, favours sarcoidosis	Negative	Negative	NA	NA

Online supplements are not copyedited prior to posting and the author(s) take full responsibility for the accuracy of all data.

**T1**	63	Male	Eurasian	TB?	NA	NA	NA	Epithelioid granulomas	Negative	Positive day 22	Isoniazid resistant	NA
**T2**	21	Male	African	TB?	NA	NA	NA	Necrotising granulomas	Negative	Positive day 14	Fully sensitive	NA
**T3**	25	Male	Eurasian	TB?	NA	NA	NA	Necrotising granulomatous inflammation-suggestive of TB	Negative	Positive day 36	Fully sensitive	NA
**T4**	26	Male	African	TB?	NA	NA	NA	Ill formed necrotising granulomas-suggests TB	Negative	Positive day 36	Fully sensitive	NA
**T5**	35	Male	Eurasian	TB /lymphoma?	NA	NA	NA	Collections of macrophages, no well-formed granulomas	Positive	Positive day 3	Rifampicin resistant	NA
**T6**	35	Male	Eurasian	TB?	NA	NA	NA	Necrotic material & MGC, consistent with TB	Negative	Positive day 14	Rifampicin resistant	NA
**T7**	71	Male	Eurasian	TB	NA	NA	NA	Caseating granulomas and MGC, highly suggestive of TB	Negative	Positive day 16	Fully sensitive	NA
**T8**	51	Female	Eurasian	TB?	NA	NA	NA	Necrosis only	Positive	Positive day 9	Fully sensitive	NA
**T9**	29	Male	Eurasian	TB?	NA	NA	NA	A few epithelioid macrophages suggesting granulomatous inflammation	Negative	Positive day 14	Fully sensitive	NA
**R1**	69	Male	Eurasian	Recurrent adenocarcinoma lung?	NA	NA	NA	No malignant cells or granulomas	Negative	Negative	NA	NA
**R2**	78	Male	Eurasian	Malignancy?	NA	NA	NA	Anthracotic lymph node-no granulomas, no malignant cells	Negative	Negative	NA	NA
**R3**	52	Male	Eurasian	Cause of lymphadenopathy?	NA	NA	NA	Occasional lymphoid cells, no granulomas or malignancy	Negative	Negative	NA	NA
**R4**	63	Male	Eurasian	Cause of lymphadenopathy?	NA	NA	NA	Lymphoid tissue, no malignant cells or granulomas	Negative	Negative	NA	NA
**R5**	50	Male	Eurasian	Sarcoidosis?	NA	NA	NA	Lymphoid cells, no malignancy or granulomas	Negative	Negative	NA	NA
**R6**	33	Male	Eurasian	TB?	NA	NA	NA	Scanty lymphoid cells	Negative	Negative	NA	NA
**R7**	62	Male	Eurasian	IPF?	NA	NA	NA	Lymphoid cells	Negative	Negative	NA	NA
**R8**	71	Male	Eurasian	TB?	NA	NA	NA	No malignant cells or granulomas	Negative	Negative	NA	NA
**R9**	73	Male	Eurasian	Malignancy/ reactive?	NA	NA	NA	Lymphoid cells, no malignant cells or granulomas	Negative	Negative	NA	NA
**R10**	40	Female	Eurasian	TB?	NA	NA	NA	No granulomas or malignant cells	Negative	Negative	NA	NA
**C1**	58	Male	Eurasian	Malignancy?	NA	NA	NA	Non-small cell carcinoma	Negative	Negative	NA	NA

Online supplements are not copyedited prior to posting and the author(s) take full responsibility for the accuracy of all data.

**C2**	64	Female	Eurasian	Metastatic breast cancer?	NA	NA	NA	Metastatic breast carcinoma	Negative	Negative	NA	NA	
**C3**	86	Male	Eurasian	Malignancy?	NA	NA	NA	Non-small cell carcinoma	ND	ND	NA	NA	
**C4**	55	Male	Eurasian	Lung cancer?	NA	NA	NA	Squamous carcinoma	Negative	Negative	NA	NA	
**C5**	81	Male	Eurasian	Lung cancer?	NA	NA	NA	Non-small cell carcinoma	Negative	Negative	NA	NA	
**C6**	54	Male	Eurasian	Lung cancer?	NA	NA	NA	Non-small cell lung cancer	Negative	Negative	NA	NA	
**C7**	74	Male	Eurasian	Malignancy?	NA	NA	NA	Non-small cell carcinoma	Negative	Negative	NA	NA	
**C8**	81	Female	Eurasian	Malignancy?	NA	NA	NA	Metastatic non-small cell carcinoma	Negative	Negative	NA	NA	
**C9**	65	Female	Eurasian	Lung cancer?	NA	NA	NA	Small cell lung cancer	Negative	Negative	NA	NA	
**C10**	68	Male	Eurasian	Lung cancer?	NA	NA	NA	Metastatic non-small cell lung cancer	Negative	Negative	NA	NA	
**C11**	67	Male	Eurasian	Malignancy?	NA	NA	NA	Small cell carcinoma	Negative	Negative	NA	NA	
**C12**	75	Female	Eurasian	TB/ lymphoma/ sarcoidosis?	NA	NA	NA	Small cell carcinoma	Negative	Negative	NA	NA	
**C13**	64	Male	Eurasian	Metastatic lung cancer?	NA	NA	NA	Non-small cell carcinoma	Negative	Negative	NA	NA	
**C14**	62	Female	Eurasian	Metastatic bowel cancer/lung cancer?	NA	NA	NA	Adenocarcinoma	Negative	Negative	NA	NA	
**C15**	71	Female	Eurasian	Metastatic lung cancer?	NA	NA	NA	Metastatic squamous carcinoma	ND	ND	NA	NA	
**C16**	76	Male	Eurasian	Metastatic renal cancer?	NA	NA	NA	Metastatic adenocarcinoma-clear cell	Negative	Negative	NA	NA	
**C17**	62	Male	Eurasian	Metastatic lung cancer?	NA	NA	NA	Small cell lung cancer	Negative	Negative	NA	NA	
**C18**	77	Female	Eurasian	Metastatic lung cancer?	NA	NA	NA	Metastatic squamous carcinoma	Negative	Negative	NA	NA	
**C19**	66	Female	Eurasian	Lung cancer/ metastatic pancreatic cancer?	NA	NA	NA	Adenocarcinoma, possibly metastatic from upper GI tract	Negative	Negative	NA	NA	
**C20**	69	Male	Eurasian	Lung cancer?	NA	NA	NA	Small cell lung cancer	Negative	Negative	NA	NA	
**C21**	83	Female	Eurasian	Lung cancer?	NA	NA	NA	Non-small cell lung cancer	Negative	Negative	NA	NA	
**C22**	46	Male	East Asian	Malignancy?	NA	NA	NA	Squamous carcinoma	Negative	Negative	NA	NA	

Online supplements are not copyedited prior to posting and the author(s) take full responsibility for the accuracy of all data.

**C23**	50	Male	Eurasian	Malignancy?	NA	NA	NA	Adenocarcinoma of lung	Negative	Negative	NA	NA
**C24**	49	Female	Eurasian	Malignancy?	NA	NA	NA	Adenocarcinoma	ND	ND	NA	NA
**C25**	59	Male	African	Malignancy?	NA	NA	NA	Non-small cell carcinoma-probable squamous	Negative	Negative	NA	NA
**C26**	67	Male	Eurasian	Lung cancer?	NA	NA	NA	Small cell carcinoma	Negative	Negative	NA	NA
**C27**	62	Female	Eurasian	Lung cancer/ metastatic anal cancer?	NA	NA	NA	Small cell lung cancer	Negative	Negative	NA	NA
**Possible S1**	38	Male	Latin American	Sarcoidosis?	No	Information unavailable	Information unavailable	No granulomas or malignant cells	Negative	Negative	NA	Non-granulomatous histology
**Possible S2**	50	Male	Eurasian	Sarcoidosis?	No	120	No	Lymphoid cells, no malignancy or granulomas	Negative	Negative	NA	Non-granulomatous histology
**Possible S3**	42	Male	Eurasian	Sarcoidosis?	Yes	Information unavailable	No	No significant pathology	Negative	Negative	NA	Non-granulomatous histology
**Probable T1**	35	Female	Eurasian	TB?	NA	NA	NA	Necrotising granulomas-TB most likely	Negative	Negative	NA	Mtb culture negative
**Probable T2**	30	Female	Eurasian	TB?	NA	NA	NA	N-C granulomas	Negative	Negative	NA	Mtb culture negative
**Possible C1**	56	Female	Eurasian	Metastatic endometrial cancer?	NA	NA	NA	Epithelioid macrophages forming loose granulomas	Negative	Negative	NA	Non-malignant histology
**Possible C2**	64	Female	Eurasian	Sarcoidosis / lymphoma?	NA	NA	NA	N-C granulomas	Negative	Negative	NA	Non-malignant histology
**Possible C3**	65	Male	Eurasian	Metastatic bowel cancer?	NA	NA	NA	N-C granulomas	Negative	Negative	NA	Non-malignant histology
**Possible C4**	63	Female	Eurasian	Metastatic bowel cancer?	NA	NA	NA	N-C granulomas	Negative	Negative	NA	Non-malignant histology
**Possible C5**	72	Male	Eurasian	Metastatic lung cancer?	NA	NA	NA	Anthracotic lymph node fragments, no malignancy or granulomas	Negative	Negative	NA	Non-malignant histology
**Possible C6**	69	Female	Eurasian	Cancer?	NA	NA	NA	No malignant cells or granulomas	Negative	Negative	NA	Non-malignant histology
**Possible C7**	68	Male	Eurasian	Metastatic lung cancer?	NA	NA	NA	Lymphoid tissue, no malignant cells or granulomas	Negative	Negative	NA	Non-malignant histology
**Possible C8**	79	Male	Eurasian	Metastatic bladder/ renal cancer?	NA	NA	NA	No malignant cells or granulomas	Negative	Negative	NA	Non-malignant histology
**Possible C9**	64	Female	Eurasian	Metastatic lung cancer?	NA	NA	NA	Lymphoid cells & some epithelioid histiocytes	Negative	Negative	NA	Non-malignant histology

Online supplements are not copyedited prior to posting and the author(s) take full responsibility for the accuracy of all data.

**Possible C10**	60	Female	Eurasian	Metastatic lung cancer?	NA	NA	NA	No malignant cells or granulomas	ND	ND	NA	Non-malignant histology
**Possible C11**	70	Female	Eurasian	Metastatic lung cancer?	NA	NA	NA	Lymphoid aggregates, no malignancy or granulomas	Negative	Negative	NA	Non-malignant histology
**Possible C12**	82	Male	Eurasian	Metastatic bladder cancer?	NA	NA	NA	Lymphoid cells	Negative	Negative	NA	Non-malignant histology
**U1**	48	Female	African	Sarcoidosis/TB?	No	Information unavailable	Ocular	N-C granulomas	Negative	Negative	NA	Mtb culture negative and concurrent empirical TB treatment
**U2**	40	Male	Eurasian	Sarcoidosis?	No	Information unavailable	Peripheral nerve	Lymphoid cells, no malignancy or granulomas	Negative	Negative	NA	Non-granulomatous histology and atypical clinical presentation
**U3**	51	Female	Eurasian	Sarcoidosis/ lymphoma?	No	79	Hepatic	Small collections of lymphocytes but no well -formed granulomas	Negative	Negative	NA	Non-granulomatous histology and atypical distribution of lymphadenopathy
**U4**	53	Male	Eurasian	Sarcoidosis?	No	Information unavailable	No	N-C granulomas, favours sarcoidosis	Negative	Negative	NA	No follow up information available
**U5**	44	Male	Eurasian	Sarcoidosis/TB?	No	Information unavailable	No	N-C granulomas	Negative	Negative	NA	Mtb culture negative and concurrent empirical TB treatment
**U6**	58	Male	Eurasian	Sarcoidosis/TB/ lymphoma?	No	Information unavailable	Information unavailable	No malignant cells or granulomas	Negative	Negative	NA	Non-granulomatous histology, Mtb culture negative and concurrent empirical TB treatment

Mtb=*Mycobacterium tuberculosis*, NA=Not applicable, ND=Not done, N-C=Non-caseating, MGC=Multinucleate giant cells, TB=Tuberculosis, GI=Gastrointestinal, CNS=Central nervous system, IPF=Idiopathic pulmonary fibrosis.

E-TABLE 3. DIFFERENTIALLY EXPRESSED GENES BETWEEN GRANULOMATOUS AND NON-GRANULOMATOUS LYMPH NODES.

Agilent Probe ID	RefSeq Accession	Gene Symbol	Fold difference G>NG
**A_33_P3388501**	NM_003465	CHIT1	18.9182
**A_33_P3298159**	NM_000954	PTGDS	17.8363
**A_23_P63209**	NM_181755	HSD11B1	16.4807
**A_23_P123853**	NM_006274	CCL19	12.1249
**A_23_P34744**	NM_000396	CTSK	11.2230
**A_23_P18452**	NM_002416	CXCL9	10.3703
**A_23_P137665**	NM_001276	CHI3L1	8.7118
**A_23_P26024**	NM_032413	C15orf48	8.0793
**A_23_P43107**	NM_030788	TM7SF4	6.9073
**A_23_P200138**	NM_020125	SLAMF8	6.8351
**A_23_P150583**	NM_003357	SCGB1A1	6.6271
**A_23_P257111**	NM_000507	FBP1	6.4106
**A_23_P81898**	NM_006398	UBD	6.2910
**A_33_P3285945**	NM_002989	CCL21	6.1666
**A_23_P362694**	NM_152997	C4orf7	5.7495
**A_24_P353638**	NM_021181	SLAMF7	5.6972
**A_23_P134426**	NM_001005340	GPNMB	5.5802
**A_23_P110624**	NM_001332	CTNND2	5.4703
**A_23_P60627**	NM_001141	ALOX15B	5.3963
**A_24_P286114**	NM_004172	SLC1A3	5.3126
**A_33_P3267799**	NM_006847	LILRB4	5.2214
**A_23_P44421**	NM_153692	HTRA4	5.1930
**A_23_P40174**	NM_004994	MMP9	5.1445
**A_33_P3273885**	XM_003119266		4.9571
**A_23_P137366**	NM_000491	C1QB	4.9502
**A_24_P222655**	NM_015991	C1QA	4.9017
**A_23_P120902**	NM_006498	LGALS2	4.8114
**A_23_P36397**	NM_000785	CYP27B1	4.7922
**A_24_P852756**	NM_020056	HLA-DQA2	4.7287
**A_33_P3293049**	NM_002122	HLA-DQA1	4.6308
**A_24_P109214**	NM_001645	APOC1	4.6094
**A_33_P3390172**	NM_001145271	ADAMDEC1	4.5828
**A_33_P3413989**	NM_000062	SERPING1	4.5731
**A_23_P112026**	NM_002164	IDO1	4.5030
**A_33_P3223592**	NM_000041	APOE	4.4999
**A_33_P3380383**	NM_001099221	TIFAB	4.4201
**A_33_P3405334**	NM_000405	GM2A	4.4035
**A_23_P145096**	NM_005084	PLA2G7	4.3414
**A_33_P3281985**	NM_001006658	CR2	4.3159
**A_33_P3237775**	NM_005693	NR1H3	4.2781
**A_23_P154784**	NM_033197	C20orf114	4.1287
**A_33_P3241269**	NM_001025195	CES1	4.1194

Online supplements are not copyedited prior to posting and the author(s) take full responsibility for the accuracy of all data.

**A_23_P118203**	NM_145252	ZG16B	4.0967
**A_32_P217750**	NM_002183	IL3RA	4.0091
**A_23_P113572**	NM_001770	CD19	3.8989
**A_33_P3401990**	NM_013378	VPREB3	3.8921
**A_24_P295590**	NM_032023	RASSF4	3.8393
**A_23_P74290**	NM_052942	GBP5	3.8059
**A_33_P3245238**	NM_176825	SULT1C2	3.7822
**A_32_P356316**	NM_002119	HLA-DOA	3.7462
**A_33_P3361422**	NM_000784	CYP27A1	3.7457
**A_23_P81441**	NM_130848	C5orf20	3.6350
**A_33_P3245228**	NM_130852	PLUNC	3.6342
**A_33_P3398912**	NM_017585	SLC2A6	3.6200
**A_32_P56249**	NR_038996	LOC100131733	3.6126
**A_23_P357717**	NM_021966	TCL1A	3.5753
**A_24_P370472**	NM_021983	HLA-DRB4	3.4360
**A_24_P274270**	NM_139266	STAT1	3.4349
**A_23_P14774**	NM_004390	CTSH	3.4310
**A_23_P109913**	NM_006564	CXCR6	3.4230
**A_23_P46936**	NM_000399	EGR2	3.4167
**A_23_P116898**	NM_000014	A2M	3.4068
**A_23_P258769**	NM_002121	HLA-DPB1	3.3881
**A_24_P45446**	NM_052941	GBP4	3.3560
**A_33_P3389634**	NM_001747	CAPG	3.3343
**A_23_P19510**	NM_001198858	HLA-DQB2	3.3301
**A_24_P288836**	NR_001435	HLA-DPB2	3.3090
**A_32_P351968**	NM_002118	HLA-DMB	3.3067
**A_23_P65651**	NM_004184	WARS	3.3000
**A_24_P98109**	NM_013322	SNX10	3.2884
**A_33_P3246985**	NM_022359	PDE4DIP	3.2704
**A_33_P3234277**	NM_001242524	HLA-DPA1	3.2646
**A_23_P74778**	NM_024579	C1orf54	3.2557
**A_33_P3405424**	NM_172374	IL4I1	3.2460
**A_24_P343233**	NM_002124	HLA-DRB1	3.2420
**A_23_P63390**	NM_001017986	FCGR1B	3.2140
**A_23_P55270**	NM_002988	CCL18	3.2037
**A_33_P3222947**	NM_001146336	TMEM114	3.1672
**A_23_P101407**	NM_000064	C3	3.1636
**A_33_P3383912**	NM_022555	HLA-DRB3	3.1428
**A_24_P844984**	NM_002644	PIGR	3.1408
**A_23_P164057**	NM_002404	MFAP4	3.1357
**A_23_P106922**	NM_021615	CHST6	3.1345
**A_32_P87697**	NM_019111	HLA-DRA	3.1234
**A_33_P3406567**	NM_152866	MS4A1	3.1195
**A_23_P133474**	NM_002084	GPX3	3.1185
**A_33_P3424222**	NM_002123	HLA-DQB1	3.1014
**A_23_P31725**	NM_001715	BLK	3.0899

Online supplements are not copyedited prior to posting and the author(s) take full responsibility for the accuracy of all data.

**A_23_P312851**	NM_006928	PMEL	3.0890
**A_24_P766716**	NM_001142343	CMKLR1	3.0575
**A_24_P146683**	NM_002443	MSMB	3.0563
**A_33_P3399571**	NM_004666	VNN1	3.0388
**A_33_P3224710**	NM_012252	TFEC	2.9990
**A_24_P77082**	NM_003679	KMO	2.9893
**A_24_P156490**	NM_002247	KCNMA1	2.9687
**A_19_P00317953**		XLOC_012197	2.9609
**A_24_P935986**	NM_005504	BCAT1	2.9597
**A_23_P142075**	NM_001611	ACP5	2.9586
**A_23_P45099**	NM_002125	HLA-DRB5	2.9486
**A_23_P39067**	NM_003121	SPIB	2.9411
**A_33_P3262635**	NM_177405	CECR1	2.9379
**A_23_P46871**	NM_018344	SLC29A3	2.9377
**A_23_P36753**	NM_000690	ALDH2	2.9306
**A_23_P2492**	NM_001734	C1S	2.9262
**A_23_P100660**	NM_002615	SERPINF1	2.9171
**A_23_P167328**	NM_001775	CD38	2.9017
**A_23_P99063**	NM_002345	LUM	2.8839
**A_24_P270728**	NM_001042483	NUPR1	2.8831
**A_23_P139500**	NM_030762	BHLHE41	2.8809
**A_33_P3394140**	NR_024431	LOC283050	2.8672
**A_23_P10232**	NM_017935	BANK1	2.8629
**A_23_P7827**	NM_001010919	FAM26F	2.8566
**A_23_P97860**	NM_000235	LIPA	2.8551
**A_32_P162187**	NM_000063	C2	2.8496
**A_23_P62890**	NM_002053	GBP1	2.8463
**A_33_P3372004**	NM_005849	IGSF6	2.8369
**A_33_P3263867**	NM_002562	P2RX7	2.8364
**A_23_P328740**	NR_026875	NEURL3	2.8333
**A_33_P3247042**	NM_002030	FPR3	2.8301
**A_23_P69310**	NM_003965	CCRL2	2.8142
**A_23_P50146**	NM_213602	SIGLEC15	2.7907
**A_24_P50245**	NM_006120	HLA-DMA	2.7777
**A_24_P945113**	NM_000020	ACVRL1	2.7609
**A_23_P201211**	NM_031281	FCRL5	2.7470
**A_24_P365767**	NM_000397	CYBB	2.7408
**A_23_P83098**	NM_000689	ALDH1A1	2.7387
**A_23_P39840**	NM_006634	VAMP5	2.7255
**A_23_P141505**	NM_182906	CLEC10A	2.7195
**A_23_P125977**	NM_172369	C1QC	2.7095
**A_23_P151166**	NM_001040107	HVCN1	2.7060
**A_23_P26325**	NM_002987	CCL17	2.7052
**A_23_P75786**	NM_016582	SLC15A3	2.7034
**A_23_P62647**	NM_003037	SLAMF1	2.6995
**A_32_P221305**	NR_024420	LOC389634	2.6978

Online supplements are not copyedited prior to posting and the author(s) take full responsibility for the accuracy of all data.

**A_32_P30905**	NM_020945	WDFY4	2.6921
**A_23_P104798**	NM_001562	IL18	2.6873
**A_23_P207201**	NM_001039933	CD79B	2.6870
**A_33_P3244122**	NM_012205	HAAO	2.6866
**A_33_P3360972**	NM_001077594	EXOC3L4	2.6777
**A_33_P3250680**	NM_000074	CD40LG	2.6661
**A_23_P312920**	NM_006235	POU2AF1	2.6635
**A_23_P70095**	NM_001025158	CD74	2.6628
**A_23_P15394**	NM_001251	CD68	2.6482
**A_33_P3395605**	NM_181724	TMEM119	2.6449
**A_33_P3338733**	NM_198159	MITF	2.6433
**A_23_P412321**	NM_000579	CCR5	2.6376
**A_23_P70670**	NM_004233	CD83	2.6319
**A_23_P99163**	NM_018370	DRAM1	2.6307
**A_33_P3316273**	NM_002983	CCL3	2.6244
**A_33_P3343120**	NM_002163	IRF8	2.6117
**A_23_P50508**	NM_003706	PLA2G4C	2.6109
**A_23_P18078**	NM_002888	RARRES1	2.6068
**A_23_P46039**	NM_032738	FCRLA	2.6028
**A_33_P3368334**		FCRL3	2.5988
**A_23_P134347**	NM_019029	CPVL	2.5866
**A_23_P57036**	NM_001250	CD40	2.5691
**A_33_P3343175**	NM_001565	CXCL10	2.5552
**A_23_P209625**	NM_000104	CYP1B1	2.5549
**A_23_P152548**	NM_021626	SCPEP1	2.5464
**A_23_P343398**	NM_001838	CCR7	2.5364
**A_23_P64873**	NM_001920	DCN	2.5303
**A_23_P30736**	NM_002120	HLA-DOB	2.5273
**A_23_P202448**	NM_199168	CXCL12	2.5234
**A_23_P150316**	NM_002426	MMP12	2.5070
**A_33_P3255304**	NM_001099781	GGT5	2.5060
**A_33_P3248265**	NM_002341	LTB	2.5058
**A_23_P84596**	NM_016459	MZB1	2.4971
**A_33_P3379268**	NM_207103	C17orf87	2.4917
**A_24_P416997**	NM_145641	APOL3	2.4885
**A_24_P252945**	NM_032966	CXCR5	2.4878
**A_23_P157007**	NM_014020	TMEM176B	2.4852
**A_23_P151895**	NM_003613	CILP	2.4812
**A_24_P346431**	NM_022748	TNS3	2.4751
**A_23_P342131**	NM_153611	CYBASC3	2.4739
**A_23_P251881**	NM_147130	NCR3	2.4735
**A_24_P82106**	NM_004995	MMP14	2.4723
**A_23_P361940**	NM_003874	CD84	2.4709
**A_24_P365975**	NM_005202	COL8A2	2.4457
**A_33_P3287631**	NM_147780	CTSB	2.4386
**A_23_P11543**	NM_000147	FUCA1	2.4342

Online supplements are not copyedited prior to posting and the author(s) take full responsibility for the accuracy of all data.

**A_23_P212655**	NM_130446	KLHL6	2.4257
**A_33_P3364180**	NM_152536	FGD5	2.4056
**A_23_P134237**	NM_002889	RARRES2	2.3992
**A_23_P89981**	NM_000774	CYP2F1	2.3986
**A_23_P167168**	NM_144646	IGJ	2.3900
**A_24_P191588**	NM_000688	ALAS1	2.3811
**A_24_P165864**	NM_014879	P2RY14	2.3803
**A_23_P14165**	NM_005292	GPR18	2.3789
**A_23_P312132**	NM_000887	ITGAX	2.3759
**A_33_P3368313**	NM_005951	MT1H	2.3754
**A_23_P376488**	NM_000594	TNF	2.3631
**A_24_P387875**	NM_002241	KCNJ10	2.3625
**A_23_P311875**	NM_006725	CD6	2.3621
**A_23_P108948**	NM_018000	MREG	2.3540
**A_23_P421423**	NM_006291	TNFAIP2	2.3458
**A_23_P49759**	NM_002981	CCL1	2.3447
**A_32_P32254**	NM_001848	COL6A1	2.3426
**A_24_P227927**	NM_181078	IL21R	2.3395
**A_23_P12680**	NM_001042465	PSAP	2.3268
**A_32_P77102**	NR_038461	LOC100128420	2.3254
**A_23_P12746**	NM_002438	MRC1	2.3254
**A_23_P55356**	NM_182566	VMO1	2.3234
**A_23_P114299**	NM_001504	CXCR3	2.3208
**A_33_P3341105**	NM_002602	PDE6G	2.3182
**A_24_P355145**	NM_033105	DNAJC5B	2.3180
**A_33_P3243907**	NM_001909	CTSD	2.3137
**A_32_P175934**	NM_001778	CD48	2.3101
**A_24_P224727**	NM_004364	CEBPA	2.3093
**A_33_P3292769**	NM_145912	NFAM1	2.3087
**A_33_P3211432**	NM_000265	NCF1	2.3033
**A_23_P40240**	NM_001336	CTSZ	2.3017
**A_23_P101992**	NM_006770	MARCO	2.2916
**A_23_P55020**	NM_139018	CD300LF	2.2875
**A_32_P209960**	NM_000246	CIITA	2.2866
**A_23_P215913**	NM_203339	CLU	2.2821
**A_23_P217109**	NM_001860	SLC31A2	2.2785
**A_23_P9415**	NM_002197	ACO1	2.2778
**A_23_P138125**	NM_005449	FAIM3	2.2749
**A_33_P3259393**	NM_178232	HAPLN3	2.2745
**A_33_P3294177**		LOC100131043	2.2697
**A_33_P3363637**	NM_013314	BLNK	2.2601
**A_23_P140190**	NR_026800	KIAA0125	2.2518
**A_23_P107775**	NM_139172	TMEM190	2.2504
**A_24_P211044**	NM_001317	CSH1	2.2474
**A_23_P23639**	NM_153259	MCOLN2	2.2359
**A_23_P99642**	NM_001126106	SLC7A7	2.2352

Online supplements are not copyedited prior to posting and the author(s) take full responsibility for the accuracy of all data.

**A_23_P85240**	NM_016562	TLR7	2.2334
**A_23_P89570**	NM_032265	ZMYND15	2.2314
**A_23_P43369**	NM_014450	SIT1	2.2294
**A_24_P239076**	NM_020070	IGLL1	2.2235
**A_23_P354387**	NM_013451	MYOF	2.2201
**A_23_P212617**	NM_003234	TFRC	2.2194
**A_24_P319647**	NM_030764	FCRL2	2.2190
**A_33_P3351745**	NM_024070	PVRIG	2.2181
**A_23_P92042**	NM_002222	ITPR1	2.2174
**A_24_P228130**	NM_001001437	CCL3L3	2.2152
**A_33_P3234202**	NM_004944	DNASE1L3	2.2115
**A_24_P365901**	NM_178562	TSPAN33	2.2115
**A_33_P3375859**	NR_002712	CXCR2P1	2.2112
**A_23_P1552**	NM_001814	CTSC	2.2110
**A_23_P323761**	NM_025228	TRAF3IP3	2.2093
**A_23_P18282**	NM_007335	DLEC1	2.2036
**A_23_P207911**	NM_016113	TRPV2	2.2026
**A_33_P3295056**	NM_005608	PTPRCAP	2.2008
**A_23_P161076**	NM_001767	CD2	2.2004
**A_23_P160159**	NM_003039	SLC2A5	2.1958
**A_23_P329573**	NM_000211	ITGB2	2.1904
**A_23_P40611**	NM_000355	TCN2	2.1898
**A_23_P161769**	NM_021603	FXYD2	2.1894
**A_33_P3351249**	NM_001100812	CXCL16	2.1881
**A_24_P931443**	NM_003485	GPR68	2.1875
**A_33_P3379039**	NM_001178126	IGLL5	2.1875
**A_23_P41145**	NM_138805	FAM3D	2.1818
**A_23_P209678**	NM_002664	PLEK	2.1755
**A_23_P85693**	NM_004120	GBP2	2.1726
**A_23_P85800**	NM_001803	CD52	2.1717
**A_33_P3376958**	NR_027293	LOC96610	2.1617
**A_23_P255104**	NM_005779	LHFPL2	2.1579
**A_32_P171061**	NM_005170	ASCL2	2.1576
**A_23_P209055**	NM_001771	CD22	2.1535
**A_23_P103361**	NM_005356	LCK	2.1522
**A_33_P3334515**	NM_201535	NDRG2	2.1517
**A_23_P41765**	NM_002198	IRF1	2.1437
**A_23_P433785**	NM_002561	P2RX5	2.1433
**A_24_P357847**	XM_003120829	LOC100510044	2.1426
**A_23_P18604**	NM_015907	LAP3	2.1397
**A_33_P3250730**	NM_018674	ACCN4	2.1395
**A_33_P3210492**	NM_031909	C1QTNF4	2.1385
**A_23_P144877**	NM_004045	ATOX1	2.1364
**A_24_P386746**	NM_031491	RBP5	2.1356
**A_23_P250245**	NM_001782	CD72	2.1332
**A_23_P14174**	NM_006573	TNFSF13B	2.1250

Online supplements are not copyedited prior to posting and the author(s) take full responsibility for the accuracy of all data.

**A_33_P3286278**	NM_002087	GRN	2.1246
**A_23_P151294**	NM_000619	IFNG	2.1179
**A_23_P126677**	NM_021233	DNASE2B	2.1133
**A_33_P3228322**	NM_173042	IL18BP	2.1125
**A_33_P3414880**	XR_115108	LOC339192	2.1088
**A_33_P3321432**	NM_016613	FAM198B	2.1054
**A_23_P87879**	NM_001781	CD69	2.1053
**A_24_P242646**	NM_004079	CTSS	2.0964
**A_23_P208302**	NM_000483	APOC2	2.0945
**A_23_P156218**	NM_002104	GZMK	2.0919
**A_23_P30655**	NM_004556	NFKBIE	2.0914
**A_33_P3298990**	NM_014207	CD5	2.0911
**A_32_P44394**	NM_004833	AIM2	2.0903
**A_23_P315571**	NM_015150	RFTN1	2.0851
**A_32_P123255**	NM_001105576	ANKRD58	2.0836
**A_23_P99275**	NM_002258	KLRB1	2.0823
**A_33_P3231414**	NM_006669	LILRB1	2.0800
**A_23_P253317**	NM_013308	GPR171	2.0796
**A_23_P58132**	NM_004310	RHOH	2.0795
**A_33_P3423365**	NM_001127663	GSN	2.0790
**A_23_P70688**	NM_004271	LY86	2.0777
**A_23_P201778**	NM_080588	PTPN7	2.0679
**A_23_P149368**	NM_052938	FCRL1	2.0639
**A_23_P122924**	NM_002192	INHBA	2.0618
**A_33_P3816688**	NM_133263	PPARGC1B	2.0587
**A_24_P153568**	NM_001039396	MPEG1	2.0579
**A_24_P82749**	NM_001774	CD37	2.0549
**A_33_P3412900**	NM_001039771	CBLN3	2.0534
**A_33_P3383970**	NM_030956	TLR10	2.0533
**A_23_P29953**	NM_172175	IL15	2.0527
**A_23_P45475**	NM_000169	GLA	2.0514
**A_23_P37736**	NM_001192	TNFRSF17	2.0513
**A_32_P46214**	NM_173653	SLC9A9	2.0500
**A_23_P156049**	NM_000521	HEXB	2.0447
**A_32_P8813**	NR_024433	LOC283663	2.0433
**A_33_P3253144**	NM_024872	DOK3	2.0420
**A_33_P3268555**	NM_001005176	SP140	2.0396
**A_23_P349463**	NM_022097	CHP2	2.0310
**A_33_P3217776**	NM_002723	PRB4	2.0286
**A_23_P56559**	NM_005771	DHRS9	2.0275
**A_23_P360804**	NM_020939	CPNE5	2.0272
**A_23_P112452**	NR_003191	GGTA1P	2.0265
**A_24_P186379**	NM_198472	C10orf125	2.0226
**A_33_P3358923**	NM_181780	BTLA	2.0224
**A_23_P15146**	NM_001012631	IL32	2.0214
**A_23_P128974**	NM_006399	BATF	2.0186

Online supplements are not copyedited prior to posting and the author(s) take full responsibility for the accuracy of all data.

**A_23_P134176**	NM_001024465	SOD2	2.0180
**A_33_P3347343**	NM_001093729	CCDC102B	2.0175
**A_23_P165624**	NM_007115	TNFAIP6	2.0154
**A_24_P941167**	NM_030641	APOL6	2.0151
**A_23_P39814**	NM_004882	CIR1	2.0150
**A_32_P452655**	NM_001040078	LGALS9C	2.0139
**A_23_P79069**	NM_022904	RASAL3	2.0135
**A_32_P14721**	NM_178504	DNAH12	2.0119
**A_33_P3375541**	NM_000732	CD3D	2.0090
**A_23_P1962**	NM_004585	RARRES3	2.0089
**A_23_P370682**	NM_138456	BATF2	2.0077
**A_23_P128201**	NM_005337	NCKAP1L	2.0059
**A_33_P3293213**	NM_001161616	RGL3	-2.0005
**A_23_P159039**	NM_182706	SCRIB	-2.0008
**A_23_P63402**	NM_013296	GPSM2	-2.0016
**A_24_P329487**	NM_174911	FAM84B	-2.0043
**A_23_P320261**	NM_001035516	DMKN	-2.0056
**A_24_P298174**	NM_006807	CBX1	-2.0072
**A_33_P3419190**	NM_001657	AREG	-2.0091
**A_23_P18798**	NM_019119	PCDHB9	-2.0096
**A_32_P171328**	NM_014501	UBE2S	-2.0122
**A_23_P104651**	NM_080668	CDCA5	-2.0130
**A_23_P48175**	NM_024056	TMEM106C	-2.0131
**A_32_P117354**	NM_014988	LIMCH1	-2.0141
**A_23_P167401**	NM_018931	PCDHB11	-2.0180
**A_23_P250607**	NM_005032	PLS3	-2.0240
**A_23_P25305**	NM_004316	ASCL1	-2.0261
**A_24_P15621**	NR_003083	SLC6A10P	-2.0263
**A_33_P3308105**	NM_003878	GGH	-2.0322
**A_23_P113005**	NM_004428	EFNA1	-2.0328
**A_33_P3214665**	NM_002374	MAP2	-2.0368
**A_23_P104188**	NM_004433	ELF3	-2.0544
**A_23_P88522**	NM_021077	NMB	-2.0547
**A_24_P125871**	NM_020639	RIPK4	-2.0560
**A_23_P393051**	NM_152365	C1orf172	-2.0577
**A_23_P29257**	NM_005318	H1F0	-2.0586
**A_24_P693461**	NM_001101341	SFTA3	-2.0603
**A_23_P100220**	NM_024939	ESRP2	-2.0621
**A_24_P224488**	NM_016835	MAPT	-2.0640
**A_23_P136347**	NM_004447	EPS8	-2.0644
**A_32_P101031**	NM_144586	LYPD1	-2.0651
**A_23_P132718**	NM_004636	SEMA3B	-2.0672
**A_23_P119943**	NM_000597	IGFBP2	-2.0703
**A_33_P3233871**	NM_000505	F12	-2.0706
**A_23_P301846**	NM_001033952	CALCA	-2.0709
**A_23_P156284**	NM_080881	DBN1	-2.0734

Online supplements are not copyedited prior to posting and the author(s) take full responsibility for the accuracy of all data.

**A_23_P215634**	NM_001013398	IGFBP3	-2.0782
**A_33_P3393801**	NM_005764	PDZK1IP1	-2.0835
**A_23_P115482**	NM_014176	UBE2T	-2.0905
**A_24_P90216**	NM_018490	LGR4	-2.0907
**A_23_P132956**	NM_004181	UCHL1	-2.0961
**A_23_P45524**	NM_014380	NGFRAP1	-2.1004
**A_33_P3257678**	NM_001005464	HIST2H3A	-2.1022
**A_33_P3387616**	NM_052924	RHPN1	-2.1041
**A_23_P69179**	NM_018192	LEPREL1	-2.1059
**A_33_P3378126**	NM_058229	FBXO32	-2.1077
**A_33_P3237359**	NM_005342	HMGB3	-2.1108
**A_33_P3295550**	NM_030625	TET1	-2.1109
**A_23_P55251**	NM_002204	ITGA3	-2.1169
**A_33_P3303372**	NM_001184792	PARD3	-2.1240
**A_23_P148255**	NM_153488	MAGEA2B	-2.1245
**A_23_P161659**	NM_020826	SYT13	-2.1249
**A_23_P134085**	NM_173515	CNKSR3	-2.1256
**A_24_P235266**	NM_001001555	GRB10	-2.1268
**A_23_P135381**	NM_001003845	SP5	-2.1308
**A_33_P3419785**	NM_004052	BNIP3	-2.1350
**A_32_P62963**	NR_029392	KRT16P2	-2.1353
**A_23_P150935**	NM_005480	TROAP	-2.1407
**A_23_P148088**	NM_000509	FGG	-2.1413
**A_23_P420551**	NM_007174	CIT	-2.1423
**A_23_P31399**	NM_000305	PON2	-2.1498
**A_33_P3376249**	NM_005978	S100A2	-2.1505
**A_23_P114670**	NM_014448	ARHGEF16	-2.1576
**A_23_P49338**	NM_016639	TNFRSF12A	-2.1602
**A_23_P304682**	NM_001424	EMP2	-2.1615
**A_23_P210690**	NM_021158	TRIB3	-2.1619
**A_33_P3342375**	NM_175868	MAGEA6	-2.1654
**A_24_P80204**	NM_005434	MALL	-2.1690
**A_33_P3314276**	NM_181718	ASPHD1	-2.1712
**A_23_P168556**	NM_004603	STX1A	-2.1717
**A_23_P119095**	NM_006663	PPP1R13L	-2.1745
**A_23_P107421**	NM_003258	TK1	-2.1860
**A_23_P153301**	NM_004363	CEACAM5	-2.1934
**A_24_P12401**	NM_001025366	VEGFA	-2.2015
**A_23_P159952**	NM_018476	BEX1	-2.2138
**A_23_P206059**	NM_003981	PRC1	-2.2181
**A_24_P403561**	NM_002334	LRP4	-2.2275
**A_23_P312150**	NM_001956	EDN2	-2.2277
**A_24_P67681**	XM_003119674	LOC100508670	-2.2293
**A_33_P3240353**	NM_017767	SLC39A4	-2.2298
**A_23_P50426**	NM_015493	KANK2	-2.2412
**A_23_P401**	NM_016343	CENPF	-2.2412

Online supplements are not copyedited prior to posting and the author(s) take full responsibility for the accuracy of all data.

**A_33_P3321293**	NM_178229	IQGAP3	-2.2432
**A_33_P3392405**	NM_207373	C10orf99	-2.2434
**A_32_P198731**	NM_001142651	NEURL1B	-2.2602
**A_23_P20022**	NM_013332	C7orf68	-2.2669
**A_23_P127948**	NM_001124	ADM	-2.2764
**A_24_P162373**	NM_001206998	ZNRF3	-2.2766
**A_23_P363316**	NM_002147	HOXB5	-2.2799
**A_33_P3317523**	NM_203401	STMN1	-2.2914
**A_23_P373119**	NR_002165	HMGB3P1	-2.2927
**A_32_P62863**	NM_014575	SCHIP1	-2.2954
**A_23_P11800**	NM_018584	CAMK2N1	-2.3039
**A_33_P3256391**	NM_139161	CRB3	-2.3065
**A_33_P3293164**	NM_001275	CHGA	-2.3091
**A_33_P3262575**	NM_018842	BAIAP2L1	-2.3183
**A_23_P101131**	NM_002091	GRP	-2.3230
**A_23_P214950**	NM_022121	PERP	-2.3239
**A_23_P145644**	NM_000790	DDC	-2.3370
**A_33_P3318581**	NM_182943	PLOD2	-2.3384
**A_23_P214079**	NM_003122	SPINK1	-2.3552
**A_23_P118815**	NM_001012271	BIRC5	-2.3567
**A_23_P397293**	NM_017527	LY6K	-2.3595
**A_23_P50081**	NM_014214	IMPA2	-2.3628
**A_33_P3229953**	NM_001958	EEF1A2	-2.3629
**A_23_P109322**	NM_006198	PCP4	-2.3783
**A_33_P3329078**	NM_000559	HBG1	-2.3871
**A_23_P115261**	NM_000029	AGT	-2.3874
**A_33_P3260430**	NM_005988	SPRR2A	-2.3959
**A_32_P6015**	NM_005515	MNX1	-2.4063
**A_23_P417942**	NM_001024948	FNBP1L	-2.4242
**A_23_P131935**	NM_017671	FERMT1	-2.4362
**A_24_P297539**	NM_181803	UBE2C	-2.4370
**A_23_P371824**	NM_020127	TUFT1	-2.4405
**A_33_P3411628**	NM_000077	CDKN2A	-2.4522
**A_23_P108751**	NM_001039492	FHL2	-2.4681
**A_23_P124619**	NM_020672	S100A14	-2.4726
**A_33_P3240328**	NM_002653	PITX1	-2.5059
**A_33_P3403132**	NM_005234	NR2F6	-2.5103
**A_33_P3363245**	NM_007224	NXPH4	-2.5135
**A_33_P3301709**	NM_001098722	GNG4	-2.5677
**A_23_P36658**	NM_145791	MGST1	-2.5720
**A_23_P48596**	NM_198232	RNASE1	-2.5742
**A_33_P3211929**	NM_173587	RCOR2	-2.5755
**A_23_P130194**	NM_006907	PYCR1	-2.5826
**A_33_P3295523**	NM_005052	RAC3	-2.5915
**A_23_P252306**	NM_002165	ID1	-2.5916
**A_24_P61490**	NM_003317	NKX2-1	-2.6056

Online supplements are not copyedited prior to posting and the author(s) take full responsibility for the accuracy of all data.

**A_32_P96036**	NM_001093725	MEX3A	-2.6087
**A_23_P373708**	NR_028334	KRT18P55	-2.6708
**A_33_P3265359**	NM_018645	HES6	-2.6789
**A_33_P3230219**	NM_033504	TMEM54	-2.6917
**A_32_P231617**	NM_014220	TM4SF1	-2.6955
**A_33_P3244283**	NM_033259	CAMK2N2	-2.7158
**A_23_P46470**	NM_018948	ERRFI1	-2.7304
**A_33_P3329088**	NM_002773	PRSS8	-2.7430
**A_33_P3423949**	NM_005189	CBX2	-2.7845
**A_24_P414999**	NM_018407	LAPTM4B	-2.7872
**A_23_P146456**	NM_001333	CTSL2	-2.7884
**A_33_P3323847**	NM_004260	RECQL4	-2.7970
**A_24_P42136**	NM_000224	KRT18	-2.8446
**A_33_P3230698**	NM_004321	KIF1A	-2.8622
**A_23_P156970**	NM_002402	MEST	-2.8942
**A_33_P3285545**	NM_001305	CLDN4	-2.9752
**A_33_P3402565**	NM_004415	DSP	-3.0694
**A_23_P359245**	NM_000245	MET	-3.0771
**A_23_P60130**	NM_052886	MAL2	-3.1731
**A_24_P271696**	NM_001097592	XAGE1A	-3.2092
**A_33_P3317628**	NM_007183	PKP3	-3.2485
**A_33_P3292886**	NM_005554	KRT6A	-3.2774
**A_33_P3295358**	NM_139314	ANGPTL4	-3.2784
**A_33_P3587376**	NR_024214	SNAR-A3	-3.3010
**A_33_P3276703**	NM_003378	VGF	-3.4778
**A_23_P66682**	NM_018952	HOXB6	-3.4788
**A_33_P3397865**	NM_003283	TNNT1	-3.5029
**A_23_P77493**	NM_006086	TUBB3	-3.7181
**A_23_P10194**	NM_201575	SEZ6L2	-3.7404
**A_23_P91081**	NM_002354	EPCAM	-3.9569
**A_33_P3275878**	NM_000542	SFTPB	-4.1734
**A_33_P3389286**	NM_006142	SFN	-4.6883

G=Granulomatous, NG=Non-granulomatous.

Online supplements are not copyedited prior to posting and the author(s) take full responsibility for the accuracy of all data.

E-TABLE 4. DIFFERENTIALLY EXPRESSED GENES BETWEEN SARCOIDOSIS AND TUBERCULOSIS LYMPH NODES.

Agilent Probe ID	RefSeq Accession	Gene Symbol	Fold difference S>TB
**A_33_P3388501**	NM_003465	CHIT1	7.2469
**A_23_P34744**	NM_000396	CTSK	3.3119
**A_23_P132139**	NM_058180	C21orf58	2.4535
**A_33_P3235204**	NM_032213	ELMOD3	2.3929
**A_23_P43107**	NM_030788	TM7SF4	2.3773
**A_33_P3285945**	NM_002989	CCL21	2.3443
**A_33_P3288649**	NM_018951	HOXA10	2.2544
**A_33_P3222947**	NM_001146336	TMEM114	2.2083
**A_23_P50146**	NM_213602	SIGLEC15	2.1576
**A_23_P60627**	NM_001141	ALOX15B	2.1442
**A_33_P3339276**	NM_017888	ACSM5	2.0869
**A_33_P3364308**		OTOA	2.0857
**A_33_P3298159**	NM_000954	PTGDS	2.0789
**A_23_P137665**	NM_001276	CHI3L1	2.0575
**A_23_P208482**	NM_001144904	CLEC4M	2.0410
**A_23_P76622**	NM_001922	DCT	2.0025
**A_24_P28722**	NM_080657	RSAD2	-2.0233
**A_23_P207564**	NM_002984	CCL4	-2.0246
**A_33_P3423551**	NM_003897	IER3	-2.0422
**A_23_P86470**	NM_003956	CH25H	-2.0493
**A_24_P101642**	XM_001718104	LOC401847	-2.0525
**A_24_P239076**	NM_020070	IGLL1	-2.0703
**A_24_P257416**	NM_002089	CXCL2	-2.0752
**A_24_P203308**	NM_182489	STRA8	-2.0804
**A_33_P3342628**	NM_021170	HES4	-2.1024
**A_23_P127948**	NM_001124	ADM	-2.1529
**A_33_P3364869**		NAMPT	-2.2465
**A_33_P3376958**	NR_027293	LOC96610	-2.2543
**A_24_P270460**	NM_005532	IFI27	-2.2664
**A_23_P214080**	NM_001964	EGR1	-2.2769
**A_23_P19333**	NM_018643	TREM1	-2.2776
**A_24_P357847**	XM_003120829	LOC100510044	-2.3323
**A_23_P43164**	NM_015170	SULF1	-2.3356
**A_23_P45871**	NM_006820	IFI44L	-2.3892
**A_33_P3330264**	NM_001511	CXCL1	-2.4108
**A_33_P3283611**	NM_001549	IFIT3	-2.4202
**A_23_P118392**	NM_016084	RASD1	-2.4238
**A_23_P122924**	NM_002192	INHBA	-2.4244
**A_33_P3295358**	NM_139314	ANGPTL4	-2.4248
**A_23_P110712**	NM_004417	DUSP1	-2.4560
**A_23_P166408**	NM_020530	OSM	-2.7555
**A_23_P79518**	NM_000576	IL1B	-2.8329
**A_33_P3246833**	NM_173843	IL1RN	-2.8380

Online supplements are not copyedited prior to posting and the author(s) take full responsibility for the accuracy of all data.

**A_23_P64721**	NM_006018	HCAR3	-2.8455
**A_33_P3397763**	NM_003811	TNFSF9	-3.0382
**A_33_P3398331**	NM_006690	MMP24	-3.0650
**A_23_P46429**	NM_001554	CYR61	-3.1678
**A_23_P317760**	NM_173563	C6orf146	-3.2120
**A_24_P106953**	NM_025072	PTGES2	-3.2573
**A_23_P106194**	NM_005252	FOS	-3.4666
**A_33_P3405193**		C16orf72	-3.4707
**A_23_P39774**	NM_006857	SNRNP27	-3.7641
**A_23_P105012**	NM_017878	HRASLS2	-3.9505
**A_33_P3374718**	NR_033805	LOC220906	-3.9931
**A_24_P314534**	NM_181621	KRTAP13-2	-4.2549
**A_33_P3344861**	XR_108709	LOC389602	-4.7514
**A_33_P3398932**		MBD1	-5.0875
**A_33_P3252221**	NM_080723	NRSN1	-6.1697

S=Sarcoidosis, TB=Tuberculosis.

E-TABLE 5. DIFFERENTIALLY EXPRESSED GENES BETWEEN MALIGNANT AND REACTIVE LYMPH NODES.

Agilent Probe ID	RefSeq Accession	Gene Symbol	Fold difference C>R
**A_33_P3389286**	NM_006142	SFN	13.3613
**A_23_P91081**	NM_002354	EPCAM	12.8053
**A_23_P70398**	NM_001025370	VEGFA	10.2713
**A_33_P3329088**	NM_002773	PRSS8	10.1622
**A_33_P3295358**	NM_139314	ANGPTL4	10.0319
**A_23_P7313**	NM_001040058	SPP1	9.9893
**A_24_P131589**	NM_006889	CD86	8.8216
**A_24_P180680**	NM_018407	LAPTM4B	8.7048
**A_33_P3587376**	NR_024214	SNAR-A3	8.4805
**A_23_P66798**	NM_002276	KRT19	8.4713
**A_24_P385313**	NM_002840	PTPRF	8.3157
**A_23_P60130**	NM_052886	MAL2	8.0406
**A_23_P359245**	NM_000245	MET	7.7515
**A_24_P18802**	NM_020857	VPS18	7.7154
**A_33_P3390057**	NM_014220	TM4SF1	7.6132
**A_33_P3317628**	NM_007183	PKP3	7.2628
**A_23_P10194**	NM_201575	SEZ6L2	7.2151
**A_23_P36658**	NM_145791	MGST1	7.2115
**A_24_P932736**		HMBOX1	7.1982
**A_33_P3318581**	NM_182943	PLOD2	7.0399
**A_23_P214950**	NM_022121	PERP	6.8068
**A_19_P00800206**		XLOC_002130	6.6630
**A_23_P24716**	NM_017870	TMEM132A	6.5414
**A_32_P157945**	NM_004415	DSP	6.4422
**A_23_P119448**	NM_014931	PPP6R1	6.3886
**A_19_P00802433**		XLOC_005327	6.2772
**A_33_P3285545**	NM_001305	CLDN4	6.0775
**A_33_P3389827**	NM_001165978	PROM2	6.0763
**A_33_P3338121**	NM_001017402	LAMB3	5.9301
**A_33_P3347417**	NM_015001	SPEN	5.9266
**A_33_P3230219**	NM_033504	TMEM54	5.8511
**A_23_P46470**	NM_018948	ERRFI1	5.8497
**A_24_P42136**	NM_000224	KRT18	5.8420
**A_23_P77493**	NM_006086	TUBB3	5.6714
**A_24_P335092**	NM_000331	SAA1	5.6130
**A_23_P373708**	NR_028334	KRT18P55	5.6118
**A_23_P334709**	NM_007270	FKBP9	5.5671
**A_23_P130194**	NM_006907	PYCR1	5.4663
**A_24_P924862**	NM_213589	RAPH1	5.3487
**A_23_P252306**	NM_002165	ID1	5.2969
**A_33_P3292886**	NM_005554	KRT6A	5.2917
**A_23_P137856**	NM_002456	MUC1	5.2826
**A_33_P3247624**	NM_001029874	REP15	5.2656

Online supplements are not copyedited prior to posting and the author(s) take full responsibility for the accuracy of all data.

**A_23_P39034**	NM_003072	SMARCA4	5.0748
**A_33_P3275878**	NM_000542	SFTPB	4.9650
**A_33_P3304668**	NM_000088	COL1A1	4.9182
**A_23_P116235**	NM_001012334	MDK	4.9147
**A_23_P373119**	NR_002165	HMGB3P1	4.8690
**A_23_P20494**	NM_006096	NDRG1	4.8532
**A_32_P105549**	NM_001630	ANXA8L2	4.8392
**A_23_P49338**	NM_016639	TNFRSF12A	4.8292
**A_23_P106682**	NM_001424	EMP2	4.8017
**A_33_P3376249**	NM_005978	S100A2	4.7584
**A_33_P3397865**	NM_003283	TNNT1	4.7455
**A_23_P155463**	NM_024512	LRRC2	4.7197
**A_33_P3303372**	NM_001184792	PARD3	4.6995
**A_33_P3240328**	NM_002653	PITX1	4.6985
**A_33_P3236416**	NM_001004334	GPR179	4.6936
**A_23_P66682**	NM_018952	HOXB6	4.6831
**A_23_P410965**	NM_020888	KIAA1522	4.6537
**A_23_P69537**	NM_006681	NMU	4.6269
**A_23_P254212**	NM_013347	RPA4	4.5095
**A_33_P3323847**	NM_004260	RECQL4	4.4977
**A_33_P3393341**	NM_022896	LPIN3	4.4967
**A_23_P108751**	NM_001039492	FHL2	4.4891
**A_23_P146456**	NM_001333	CTSL2	4.4884
**A_24_P67681**	XM_003119674	LOC100508670	4.4640
**A_24_P270460**	NM_005532	IFI27	4.4163
**A_23_P124619**	NM_020672	S100A14	4.4122
**A_33_P3228460**	NM_001136007	FXYD3	4.4028
**A_33_P3403132**	NM_005234	NR2F6	4.3865
**A_23_P150316**	NM_002426	MMP12	4.3768
**A_23_P119943**	NM_000597	IGFBP2	4.3653
**A_33_P3243405**	NM_007264	GPR182	4.3650
**A_32_P108655**	NM_001005353	AK4	4.3399
**A_23_P26024**	NM_032413	C15orf48	4.3036
**A_23_P20022**	NM_013332	C7orf68	4.2852
**A_23_P127948**	NM_001124	ADM	4.2362
**A_23_P104188**	NM_004433	ELF3	4.1840
**A_23_P62932**	NM_001677	ATP1B1	4.1571
**A_33_P3226167**	NM_022833	FAM129B	4.1478
**A_33_P3229107**	NM_001104548	LOC642587	4.1461
**A_24_P125871**	NM_020639	RIPK4	4.1251
**A_23_P206280**	NM_201525	GPR56	4.1206
**A_23_P371824**	NM_020127	TUFT1	4.1183
**A_24_P61490**	NM_003317	NKX2-1	4.1153
**A_23_P149529**	NM_002353	TACSTD2	4.1136
**A_24_P271696**	NM_001097592	XAGE1A	4.1023
**A_23_P166306**	NM_000071	CBS	4.0965

Online supplements are not copyedited prior to posting and the author(s) take full responsibility for the accuracy of all data.

CHEST Online Supplement

**A_33_P3308105**	NM_003878	GGH	4.0594
**A_33_P3285565**	NM_001306	CLDN3	4.0557
**A_23_P118815**	NM_001012271	BIRC5	4.0132
**A_23_P27795**	NM_021102	SPINT2	3.9960
**A_24_P297539**	NM_181803	UBE2C	3.9878
**A_23_P43490**	NM_058197	CDKN2A	3.9747
**A_33_P3319041**	NM_005342	HMGB3	3.9709
**A_33_P3310780**	NM_005231	CTTN	3.9651
**A_23_P131935**	NM_017671	FERMT1	3.9572
**A_33_P3315263**	NM_175834	KRT79	3.9189
**A_33_P3265359**	NM_018645	HES6	3.9158
**A_33_P3222917**	NM_001024736	CD276	3.9082
**A_33_P3423949**	NM_005189	CBX2	3.8924
**A_32_P164246**	NM_033260	FOXQ1	3.8799
**A_23_P47565**	NM_005566	LDHA	3.8617
**A_23_P156970**	NM_002402	MEST	3.8454
**A_33_P3508822**	NM_000484	APP	3.8379
**A_19_P00805812**		XLOC_013282	3.8227
**A_23_P52761**	NM_002423	MMP7	3.8013
**A_33_P3244283**	NM_033259	CAMK2N2	3.7937
**A_23_P417942**	NM_001024948	FNBP1L	3.7929
**A_23_P214079**	NM_003122	SPINK1	3.7889
**A_32_P96036**	NM_001093725	MEX3A	3.7889
**A_33_P3293913**	NM_001080512	BICC1	3.7690
**A_33_P3262575**	NM_018842	BAIAP2L1	3.7560
**A_33_P3269203**	NM_001207014	SERPINH1	3.7546
**A_33_P3344831**	NM_018004	TMEM45A	3.7361
**A_33_P3276703**	NM_003378	VGF	3.7328
**A_23_P397293**	NM_017527	LY6K	3.7304
**A_23_P166686**	NM_016201	AMOTL2	3.7301
**A_23_P320261**	NM_001035516	DMKN	3.7150
**A_23_P208293**	NM_001042724	PVRL2	3.7071
**A_33_P3369034**	NM_001170714	BCAR1	3.7028
**A_23_P126075**	NM_002245	KCNK1	3.6965
**A_23_P31399**	NM_000305	PON2	3.6896
**A_23_P401**	NM_016343	CENPF	3.6823
**A_23_P159775**	NM_004961	GABRE	3.6769
**A_33_P3846653**	NR_036685	KRT19P2	3.6647
**A_23_P51397**	NM_001008493	ENAH	3.6574
**A_23_P259442**	NM_001873	CPE	3.6571
**A_24_P127235**	NM_004327	BCR	3.6492
**A_23_P206359**	NM_004360	CDH1	3.6457
**A_32_P198731**	NM_001142651	NEURL1B	3.6279
**A_23_P107421**	NM_003258	TK1	3.6049
**A_33_P3234580**	NM_000050	ASS1	3.6016
**A_23_P379475**	NM_014762	DHCR24	3.5989

Online supplements are not copyedited prior to posting and the author(s) take full responsibility for the accuracy of all data.

**A_23_P206371**	NM_003946	NOL3	3.5893
**A_23_P48550**	NM_015005	KIAA0284	3.5818
**A_24_P277934**	NM_000089	COL1A2	3.5807
**A_23_P250607**	NM_005032	PLS3	3.5796
**A_23_P52727**	NM_182964	NAV2	3.5764
**A_23_P134085**	NM_173515	CNKSR3	3.5756
**A_33_P3211929**	NM_173587	RCOR2	3.5717
**A_24_P80204**	NM_005434	MALL	3.5532
**A_33_P3230698**	NM_004321	KIF1A	3.5523
**A_33_P3295523**	NM_005052	RAC3	3.5432
**A_23_P157715**	NM_032902	PPP1R16A	3.5406
**A_33_P3359368**	NM_001082488	DHRS4L1	3.5355
**A_23_P163227**	NM_001015001	CKMT1A	3.5299
**A_33_P3215948**	NM_005797	MPZL2	3.5240
**A_23_P30363**	NM_004199	P4HA2	3.5214
**A_23_P94030**	NM_002291	LAMB1	3.5154
**A_23_P218442**	NM_002483	CEACAM6	3.5039
**A_23_P47614**	NM_003311	PHLDA2	3.4974
**A_24_P137434**	NM_080927	DCBLD2	3.4939
**A_23_P68610**	NM_012112	TPX2	3.4871
**A_32_P217655**	NR_027355	LOC645166	3.4868
**A_23_P18372**	NM_032047	B3GNT5	3.4763
**A_33_P3316878**	NM_024536	CHPF	3.4593
**A_24_P188071**	NM_032704	TUBA1C	3.4531
**A_23_P104651**	NM_080668	CDCA5	3.4465
**A_23_P148057**	NM_016032	ZDHHC9	3.4271
**A_33_P3232011**	NM_022449	RAB17	3.4112
**A_33_P3377364**	NM_000213	ITGB4	3.3925
**A_33_P3239185**	NM_004200	SYT7	3.3872
**A_23_P96158**	NM_000422	KRT17	3.3750
**A_33_P3321293**	NM_178229	IQGAP3	3.3662
**A_23_P50096**	NM_001071	TYMS	3.3540
**A_24_P36890**	NM_002885	RAP1GAP	3.3460
**A_33_P3363560**	NM_001136216	TMEM51	3.3428
**A_24_P117029**	NM_000527	LDLR	3.3304
**A_32_P62863**	NM_014575	SCHIP1	3.3237
**A_24_P693461**	NM_001101341	SFTA3	3.3078
**A_33_P3807062**	NM_018410	HJURP	3.3021
**A_23_P209904**	NM_002081	GPC1	3.2997
**A_23_P143348**	NM_021220	OVOL2	3.2983
**A_23_P24104**	NM_002658	PLAU	3.2957
**A_23_P420551**	NM_007174	CIT	3.2907
**A_23_P396981**	NM_001012506	CCDC66	3.2906
**A_24_P149036**	NM_001387	DPYSL3	3.2841
**A_23_P115482**	NM_014176	UBE2T	3.2699
**A_23_P73239**	NM_205842	NCKAP1	3.2596

Online supplements are not copyedited prior to posting and the author(s) take full responsibility for the accuracy of all data.

**A_23_P49155**	NM_001793	CDH3	3.2494
**A_23_P119095**	NM_006663	PPP1R13L	3.2399
**A_23_P206059**	NM_003981	PRC1	3.2344
**A_23_P146284**	NM_003129	SQLE	3.2306
**A_24_P90216**	NM_018490	LGR4	3.2176
**A_33_P3233645**	NM_005950	MT1G	3.2104
**A_32_P62963**	NR_029392	KRT16P2	3.2085
**A_23_P69586**	NM_005245	FAT1	3.2054
**A_23_P71480**	NM_005218	DEFB1	3.1947
**A_33_P3369844**	NM_013230	CD24	3.1806
**A_23_P46429**	NM_001554	CYR61	3.1711
**A_24_P403561**	NM_002334	LRP4	3.1674
**A_23_P81392**	NM_015238	WWC1	3.1600
**A_23_P111621**	NM_005685	GTF2IRD1	3.1530
**A_23_P501822**	NM_002230	JUP	3.1509
**A_23_P208788**	NM_033520	C19orf33	3.1469
**A_23_P203299**	NM_002901	RCN1	3.1417
**A_23_P380614**	NM_006045	ATP9A	3.1396
**A_23_P171143**	NM_003270	TSPAN6	3.1370
**A_32_P163858**	NM_005063	SCD	3.1246
**A_23_P312150**	NM_001956	EDN2	3.1171
**A_23_P205031**	NM_001846	COL4A2	3.1160
**A_24_P162373**	NM_001206998	ZNRF3	3.0973
**A_23_P158925**	NM_145290	GPR125	3.0899
**A_33_P3398331**	NM_006690	MMP24	3.0879
**A_33_P3246418**	NM_005586	MDFI	3.0841
**A_33_P3408918**	NM_030754	SAA2	3.0713
**A_23_P16944**	NM_001006946	SDC1	3.0691
**A_23_P109034**	NM_002999	SDC4	3.0686
**A_23_P115091**	NM_020387	RAB25	3.0685
**A_33_P3210203**	NM_032451	SPIRE2	3.0662
**A_23_P94422**	NM_014791	MELK	3.0635
**A_23_P13899**	NM_002046	GAPDH	3.0625
**A_32_P117354**	NM_014988	LIMCH1	3.0523
**A_33_P3236881**	NM_001204088	C1orf15-NBL1	3.0462
**A_33_P3242543**	NM_000240	MAOA	3.0388
**A_33_P3229196**	NM_004357	CD151	3.0381
**A_23_P52207**	NM_012342	BAMBI	3.0199
**A_23_P362893**	NM_021961	TEAD1	3.0153
**A_33_P3351175**	NM_006648	WNK2	3.0050
**A_23_P49060**	NM_181642	SPINT1	3.0032
**A_23_P348257**	NM_014840	NUAK1	3.0004
**A_32_P104063**	NR_034105	CRNDE	2.9993
**A_23_P76488**	NM_001423	EMP1	2.9896
**A_23_P90601**	NM_182915	STEAP3	2.9885
**A_23_P201636**	NM_005562	LAMC2	2.9882

Online supplements are not copyedited prior to posting and the author(s) take full responsibility for the accuracy of all data.

**A_23_P65757**	NM_004701	CCNB2	2.9852
**A_32_P62997**	NM_018492	PBK	2.9837
**A_23_P118834**	NM_001067	TOP2A	2.9815
**A_23_P145644**	NM_000790	DDC	2.9746
**A_23_P153301**	NM_004363	CEACAM5	2.9740
**A_24_P95154**	NM_178234	TUSC3	2.9649
**A_23_P138507**	NM_001786	CDK1	2.9646
**A_23_P213678**	NM_000919	PAM	2.9540
**A_33_P3415430**	NM_005346	HSPA1B	2.9530
**A_33_P3419785**	NM_004052	BNIP3	2.9452
**A_33_P3329974**	NM_020770	CGN	2.9439
**A_23_P363316**	NM_002147	HOXB5	2.9430
**A_24_P24685**	XM_001130734	HMGB3P22	2.9390
**A_23_P31143**	NM_001003395	TPD52L1	2.9279
**A_23_P132718**	NM_004636	SEMA3B	2.9275
**A_23_P122863**	NM_001001555	GRB10	2.9214
**A_32_P171328**	NM_014501	UBE2S	2.9126
**A_23_P97990**	NM_002775	HTRA1	2.9124
**A_33_P3309491**	NM_005704	PTPRU	2.9060
**A_23_P311150**	NM_138288	C14orf147	2.9058
**A_23_P92261**	NM_032331	ECE2	2.9028
**A_33_P3387621**	NM_033103	RHPN2	2.8917
**A_24_P190168**	NM_014573	TMEM97	2.8884
**A_33_P3349637**	NM_002587	PCDH1	2.8859
**A_33_P3363245**	NM_007224	NXPH4	2.8849
**A_23_P138168**	NM_001839	CNN3	2.8804
**A_23_P34142**	NM_016303	WBP5	2.8785
**A_23_P331928**	NM_133493	CD109	2.8753
**A_23_P122197**	NM_031966	CCNB1	2.8673
**A_23_P91850**	NM_144717	IL20RB	2.8664
**A_33_P3256391**	NM_139161	CRB3	2.8644
**A_23_P156687**	NM_001710	CFB	2.8605
**A_23_P93311**	NM_013993	DDR1	2.8604
**A_23_P402751**		COX2	2.8600
**A_32_P84009**	NM_181521	CMTM4	2.8595
**A_23_P45917**	NM_001826	CKS1B	2.8566
**A_19_P00318645**		XLOC_011950	2.8523
**A_23_P52017**	NM_018136	ASPM	2.8520
**A_23_P164284**	NM_001307	CLDN7	2.8477
**A_23_P53557**	NM_002342	LTBR	2.8473
**A_23_P11800**	NM_018584	CAMK2N1	2.8386
**A_23_P65918**	NM_002220	ITPKA	2.8331
**A_24_P303524**	NM_182924	MICALL2	2.8310
**A_23_P212617**	NM_003234	TFRC	2.8302
**A_23_P24444**	NM_001360	DHCR7	2.8289
**A_23_P207850**	NM_032865	TNS4	2.8264

Online supplements are not copyedited prior to posting and the author(s) take full responsibility for the accuracy of all data.

**A_23_P34788**	NM_006845	KIF2C	2.8236
**A_23_P89249**	NM_001005862	ERBB2	2.8206
**A_23_P201386**	NM_012137	DDAH1	2.8189
**A_33_P3317523**	NM_203401	STMN1	2.8163
**A_33_P3252043**	NM_000918	P4HB	2.8150
**A_23_P66948**	NM_022751	FAM59A	2.8105
**A_33_P3314276**	NM_181718	ASPHD1	2.8000
**A_33_P3257030**	NM_006455	LEPREL4	2.7979
**A_23_P118392**	NM_016084	RASD1	2.7973
**A_23_P129103**	NR_026811	AGSK1	2.7890
**A_33_P3393821**	NM_001733	C1R	2.7861
**A_23_P89509**	NM_006461	SPAG5	2.7835
**A_23_P80068**	NM_006806	BTG3	2.7815
**A_32_P6015**	NM_005515	MNX1	2.7812
**A_24_P350200**		COX3	2.7783
**A_23_P375**	NM_018101	CDCA8	2.7765
**A_23_P27133**	NM_002275	KRT15	2.7676
**A_23_P88740**	NM_018455	CENPN	2.7649
**A_23_P57268**	NM_001338	CXADR	2.7624
**A_23_P156284**	NM_080881	DBN1	2.7614
**A_33_P3295550**	NM_030625	TET1	2.7573
**A_23_P74115**	NM_003579	RAD54L	2.7557
**A_33_P3229617**	NM_001099401	SGCE	2.7553
**A_23_P78980**	NM_014256	B3GNT3	2.7553
**A_33_P3340025**	NM_021067	GINS1	2.7502
**A_23_P147918**	NM_080388	S100A16	2.7476
**A_23_P408095**	NM_001011546	DSTN	2.7452
**A_23_P118894**	NM_024320	PRR15L	2.7449
**A_23_P127186**	NM_206862	TACC2	2.7422
**A_23_P168556**	NM_004603	STX1A	2.7331
**A_23_P431268**	NM_014935	PLEKHA6	2.7319
**A_23_P27035**	NM_015544	TMEM98	2.7317
**A_23_P80040**	NM_006404	PROCR	2.7261
**A_33_P3410459**	NM_001204255	SCARB2	2.7256
**A_23_P114232**	NM_006406	PRDX4	2.7251
**A_23_P37127**	NM_004496	FOXA1	2.7243
**A_23_P77103**	NM_003104	SORD	2.7192
**A_23_P30666**	NM_014452	TNFRSF21	2.7164
**A_32_P107876**	NM_025074	FRAS1	2.7155
**A_23_P115261**	NM_000029	AGT	2.7126
**A_33_P3251073**	NM_178545	TMEM52	2.7119
**A_23_P160406**	NM_016121	KCTD3	2.7055
**A_24_P151582**	NM_003216	TEF	2.7014
**A_23_P372874**	NM_001024210	S100A13	2.6992
**A_23_P48561**	NM_005864	EFS	2.6969
**A_33_P3344204**	NM_024786	ZDHHC11	2.6968

Online supplements are not copyedited prior to posting and the author(s) take full responsibility for the accuracy of all data.

**A_23_P50907**	NM_002210	ITGAV	2.6923
**A_23_P68211**	NM_003124	SPR	2.6922
**A_23_P22224**	NM_004095	EIF4EBP1	2.6906
**A_33_P3334225**	NM_198839	ACACA	2.6895
**A_23_P200310**	NM_017779	DEPDC1	2.6874
**A_23_P60499**	NM_021224	ZNF462	2.6864
**A_24_P295245**	NM_032467	ASPH	2.6761
**A_23_P100220**	NM_024939	ESRP2	2.6754
**A_23_P343411**	NM_198576	AGRN	2.6674
**A_19_P00319646**		XLOC_012568	2.6658
**A_32_P25273**	NM_002156	HSPD1	2.6657
**A_23_P150935**	NM_005480	TROAP	2.6631
**A_33_P3233871**	NM_000505	F12	2.6611
**A_23_P120103**	NM_002252	KCNS3	2.6608
**A_23_P259127**	NM_017697	ESRP1	2.6579
**A_23_P53176**	NM_016725	FOLR1	2.6541
**A_33_P3337272**	NM_001004354	NRARP	2.6540
**A_23_P379614**	NM_007280	OIP5	2.6520
**A_23_P356684**	NM_018685	ANLN	2.6500
**A_23_P215549**	NM_000940	PON3	2.6484
**A_23_P118493**	NM_005486	TOM1L1	2.6430
**A_33_P3247022**	NM_057749	CCNE2	2.6426
**A_23_P106844**	NM_005953	MT2A	2.6418
**A_23_P115022**	NM_144626	TMEM125	2.6413
**A_32_P83049**	NM_014971	EFR3B	2.6352
**A_33_P3363898**	NM_001070	TUBG1	2.6295
**A_23_P106145**	NM_014584	ERO1L	2.6289
**A_23_P26557**	NM_025108	C16orf59	2.6278
**A_23_P63789**	NM_032997	ZWINT	2.6257
**A_23_P57784**	NM_021101	CLDN1	2.6227
**A_33_P3213029**	NM_017763	RNF43	2.6206
**A_23_P59261**	NM_006670	TPBG	2.6197
**A_24_P199655**	NM_138959	VANGL1	2.6184
**A_33_P3415191**	NM_005603	ATP8B1	2.6170
**A_23_P71946**	NM_017688	BSPRY	2.6158
**A_33_P3380693**	NM_207197	ADAM15	2.6133
**A_33_P3229953**	NM_001958	EEF1A2	2.6120
**A_23_P131676**	NM_020311	CXCR7	2.6102
**A_23_P115064**	NM_001878	CRABP2	2.6087
**A_33_P3297562**	NM_033267	IRX2	2.6047
**A_23_P167401**	NM_018931	PCDHB11	2.6029
**A_32_P150891**	NM_001042517	DIAPH3	2.5997
**A_23_P370097**	NM_001044385	TMEM237	2.5986
**A_23_P55251**	NM_002204	ITGA3	2.5969
**A_33_P3226832**	NM_001993	F3	2.5967
**A_23_P107963**	NM_000148	FUT1	2.5963

Online supplements are not copyedited prior to posting and the author(s) take full responsibility for the accuracy of all data.

**A_23_P94533**	NM_001912	CTSL1	2.5952
**A_23_P401675**		MARVELD2	2.5950
**A_23_P44836**	NM_022908	NT5DC2	2.5941
**A_33_P3273552**	NM_002282	KRT83	2.5893
**A_33_P3342628**	NM_021170	HES4	2.5892
**A_24_P270728**	NM_001042483	NUPR1	2.5852
**A_23_P411833**	NM_145294	WDR90	2.5843
**A_23_P398275**	NR_027001	LOC388152	2.5835
**A_23_P146512**	NM_016548	GOLM1	2.5802
**A_33_P3254946**	NM_024927	PLEKHH3	2.5794
**A_23_P58647**	NM_001903	CTNNA1	2.5759
**A_23_P157268**	NM_012129	CLDN12	2.5685
**A_23_P76364**	NM_001769	CD9	2.5675
**A_23_P69030**	NM_001850	COL8A1	2.5667
**A_23_P115872**	NM_018131	CEP55	2.5667
**A_33_P3386262**	NM_030928	CDT1	2.5659
**A_24_P218979**	NM_031299	CDCA3	2.5654
**A_23_P53891**	NM_001730	KLF5	2.5569
**A_23_P3681**	NM_018092	NETO2	2.5558
**A_23_P137391**	NM_001428	ENO1	2.5510
**A_23_P500501**	NM_000142	FGFR3	2.5433
**A_33_P3260669**	NM_006281	STK3	2.5401
**A_23_P117852**	NM_014736	KIAA0101	2.5320
**A_23_P31453**	NM_012449	STEAP1	2.5315
**A_33_P3220698**	NM_133180	EPS8L1	2.5299
**A_23_P48835**	NM_138555	KIF23	2.5291
**A_33_P3772937**		KRT8P12	2.5288
**A_24_P133253**	NM_000899	KITLG	2.5284
**A_23_P139418**	NM_198516	GALNTL4	2.5283
**A_24_P928052**	NM_003873	NRP1	2.5278
**A_23_P125265**	NM_002266	KPNA2	2.5234
**A_23_P45524**	NM_014380	NGFRAP1	2.5207
**A_23_P122924**	NM_002192	INHBA	2.5206
**A_32_P210202**	NM_203394	E2F7	2.5205
**A_23_P71727**	NM_001827	CKS2	2.5145
**A_33_P3307197**	NM_020440	PTGFRN	2.5126
**A_23_P56922**	NM_002157	HSPE1	2.5068
**A_23_P427703**	NR_001447	MT1L	2.5054
**A_24_P942068**	NM_025185	TANC2	2.5040
**A_23_P43175**	NM_144710	40422.00	2.4992
**A_23_P35066**	NM_015976	SNX7	2.4951
**A_23_P143906**	NM_022443	MLF1	2.4913
**A_33_P3350508**	NM_001080432	FTO	2.4852
**A_23_P421175**	NM_198488	FAM83H	2.4842
**A_23_P433132**	NM_173853	KRTCAP3	2.4837
**A_23_P203540**	NM_012153	EHF	2.4836

Online supplements are not copyedited prior to posting and the author(s) take full responsibility for the accuracy of all data.

**A_33_P3292854**	NM_004343	CALR	2.4835
**A_23_P315252**		ND4	2.4823
**A_24_P462899**	NM_001012507	CENPW	2.4813
**A_33_P3387616**	NM_052924	RHPN1	2.4802
**A_24_P331704**	NM_182507	KRT80	2.4794
**A_33_P3339212**	NM_004237	TRIP13	2.4779
**A_23_P215060**	NM_001018111	PODXL	2.4764
**A_33_P3262495**	NM_032772	ZNF503	2.4755
**A_23_P163481**	NM_001211	BUB1B	2.4754
**A_23_P111995**	NM_002318	LOXL2	2.4745
**A_33_P3411315**	NM_033185	KRTAP3-3	2.4710
**A_23_P48669**	NM_005192	CDKN3	2.4701
**A_33_P3397443**	NM_182687	PKMYT1	2.4684
**A_33_P3296587**	NM_000096	CP	2.4679
**A_23_P76914**	NM_005982	SIX1	2.4664
**A_23_P206724**	NM_175617	MT1E	2.4648
**A_23_P394064**	NM_012232	PTRF	2.4608
**A_23_P170186**	NM_017570	OPLAH	2.4604
**A_33_P3249872**	NM_001996	FBLN1	2.4600
**A_23_P354387**	NM_013451	MYOF	2.4592
**A_32_P95739**	NM_000365	TPI1	2.4585
**A_33_P3246007**	NM_144772	APOA1BP	2.4584
**A_23_P11629**	NM_182532	TMEM61	2.4551
**A_24_P82106**	NM_004995	MMP14	2.4538
**A_23_P385861**	NM_152562	CDCA2	2.4533
**A_23_P15727**	NM_021939	FKBP10	2.4520
**A_24_P223124**	NM_022763	FNDC3B	2.4479
**A_23_P391228**	NM_001031740	MANEAL	2.4473
**A_23_P160318**	NM_001856	COL16A1	2.4457
**A_33_P3274696**	NM_024531	GPR172A	2.4423
**A_23_P42802**	NM_004911	PDIA4	2.4421
**A_33_P3329344**	NM_004104	FASN	2.4414
**A_23_P151150**	NM_202002	FOXM1	2.4391
**A_23_P62081**	NM_003020	SCG5	2.4382
**A_24_P911676**	NM_003107	SOX4	2.4362
**A_23_P152858**	NM_018405	C17orf79	2.4357
**A_23_P152804**	NM_198175	NME1	2.4355
**A_32_P34444**	NM_025135	FHOD3	2.4353
**A_33_P3313055**	NM_000435	NOTCH3	2.4328
**A_33_P3240702**	NM_002894	RBBP8	2.4318
**A_23_P139486**	NM_004642	CDK2AP1	2.4255
**A_33_P3280094**	NM_024036	LRFN4	2.4251
**A_33_P3257678**	NM_001005464	HIST2H3A	2.4237
**A_23_P128323**	NM_001038	SCNN1A	2.4228
**A_23_P136347**	NM_004447	EPS8	2.4225
**A_23_P44974**	NM_014078	MRPL13	2.4224

Online supplements are not copyedited prior to posting and the author(s) take full responsibility for the accuracy of all data.

**A_23_P20743**	NM_032342	C9orf125	2.4208
**A_23_P73012**	NM_032823	C9orf3	2.4183
**A_23_P50426**	NM_015493	KANK2	2.4153
**A_33_P3238166**	NM_012293	PXDN	2.4129
**A_23_P130343**	NM_198991	KCTD1	2.4104
**A_33_P3332081**	NM_006558	KHDRBS3	2.4082
**A_33_P3350056**	NM_005952	MT1X	2.4054
**A_24_P303091**	NM_001565	CXCL10	2.4050
**A_23_P57306**	NM_005441	CHAF1B	2.4044
**A_23_P131866**	NM_198433	AURKA	2.4033
**A_23_P59950**	NM_015359	SLC39A14	2.4017
**A_23_P150693**	NM_014344	FJX1	2.4014
**A_23_P17065**	NM_004591	CCL20	2.4002
**A_23_P162171**	NM_006500	MCAM	2.3989
**A_23_P571**	NM_006516	SLC2A1	2.3972
**A_33_P3287646**	NM_001540	HSPB1	2.3967
**A_23_P111888**	NM_138455	CTHRC1	2.3947
**A_23_P323751**	NM_030919	FAM83D	2.3936
**A_23_P7636**	NM_004219	PTTG1	2.3916
**A_24_P358328**	NR_002187	TPI1P2	2.3914
**A_23_P68487**	NM_001719	BMP7	2.3913
**A_23_P74349**	NM_145697	NUF2	2.3907
**A_23_P117933**	NM_004483	GCSH	2.3891
**A_23_P209200**	NM_001238	CCNE1	2.3875
**A_23_P50081**	NM_014214	IMPA2	2.3810
**A_24_P129632**	NM_004747	DLG5	2.3788
**A_33_P3240353**	NM_017767	SLC39A4	2.3778
**A_23_P9293**	NM_004817	TJP2	2.3777
**A_23_P33364**	NM_001009555	SH3D19	2.3742
**A_33_P3408762**	NM_005572	LMNA	2.3736
**A_23_P127579**	NM_000317	PTS	2.3735
**A_23_P501010**	NM_000494	COL17A1	2.3729
**A_23_P43164**	NM_015170	SULF1	2.3720
**A_33_P3296497**		PTPRK	2.3718
**A_23_P159039**	NM_182706	SCRIB	2.3706
**A_23_P162874**	NM_005348	HSP90AA1	2.3683
**A_23_P503233**	NM_080738	EDARADD	2.3681
**A_23_P422724**	NM_000943	PPIC	2.3671
**A_23_P32903**	NM_000276	OCRL	2.3669
**A_23_P37983**	NM_005947	MT1B	2.3648
**A_23_P359655**	NM_152437	ZNF664	2.3640
**A_33_P3332130**	NM_006667	PGRMC1	2.3629
**A_24_P551842**		CYTB	2.3626
**A_23_P37892**	NM_133443	GPT2	2.3620
**A_23_P137532**	NM_000302	PLOD1	2.3617
**A_23_P19712**	NM_015895	GMNN	2.3609

Online supplements are not copyedited prior to posting and the author(s) take full responsibility for the accuracy of all data.

**A_23_P18798**	NM_019119	PCDHB9	2.3606
**A_23_P78543**	NM_005498	AP1M2	2.3601
**A_23_P254733**	NM_024629	MLF1IP	2.3579
**A_23_P70007**	NM_012484	HMMR	2.3579
**A_33_P3350488**	NM_016359	NUSAP1	2.3564
**A_33_P3315314**	NM_005951	MT1H	2.3558
**A_23_P5301**	NM_014553	TFCP2L1	2.3545
**A_23_P208880**	NM_013282	UHRF1	2.3540
**A_23_P29257**	NM_005318	H1F0	2.3537
**A_23_P320250**	NM_025109	MYO19	2.3519
**A_33_P3413962**	NM_001242339	PFKP	2.3473
**A_23_P48175**	NM_024056	TMEM106C	2.3471
**A_23_P153441**	NM_013312	HOOK2	2.3448
**A_24_P206047**	NM_001151	SLC25A4	2.3438
**A_23_P476**	NM_003953	MPZL1	2.3414
**A_23_P91619**	NM_002415	MIF	2.3411
**A_23_P256312**	NM_002447	MST1R	2.3390
**A_33_P3217238**	NM_014109	ATAD2	2.3389
**A_23_P30254**	NM_006622	PLK2	2.3387
**A_23_P10121**	NM_003012	SFRP1	2.3378
**A_23_P50108**	NM_006101	NDC80	2.3375
**A_32_P175739**	NM_000189	HK2	2.3345
**A_23_P370989**	NM_005914	MCM4	2.3345
**A_33_P3378126**	NM_058229	FBXO32	2.3338
**A_23_P57667**	NM_032242	PLXNA1	2.3333
**A_23_P110941**	NM_001512	GSTA4	2.3327
**A_23_P94795**	NM_003213	TEAD4	2.3299
**A_23_P99927**	NM_016395	PTPLAD1	2.3286
**A_24_P339611**	NM_004708	PDCD5	2.3282
**A_24_P130041**	NM_000786	CYP51A1	2.3260
**A_23_P138725**	NM_031484	MARVELD1	2.3245
**A_32_P129752**	NM_001017970	TMEM30B	2.3230
**A_23_P10385**	NM_016448	DTL	2.3226
**A_23_P360316**	NM_000149	FUT3	2.3221
**A_33_P3219785**	NM_023016	ANKRD57	2.3210
**A_33_P3270863**	NM_000379	XDH	2.3199
**A_33_P3410700**		ATP8	2.3175
**A_23_P148475**	NM_012310	KIF4A	2.3133
**A_23_P19182**	NM_016606	REEP2	2.3100
**A_23_P337726**		ATP6	2.3090
**A_33_P3386242**	NM_006314	CNKSR1	2.3079
**A_23_P153964**	NM_002193	INHBB	2.3018
**A_23_P392384**	NM_001185095	AIF1L	2.3009
**A_33_P3210085**	NM_001047160	NET1	2.2983
**A_33_P3881056**	XM_003119710		2.2982
**A_23_P100632**	NM_001002033	HN1	2.2975

Online supplements are not copyedited prior to posting and the author(s) take full responsibility for the accuracy of all data.

**A_23_P113613**	NM_022842	CDCP1	2.2971
**A_23_P37191**	NM_002797	PSMB5	2.2951
**A_23_P38677**	NM_006553	SLMO1	2.2922
**A_24_P12626**	NM_001753	CAV1	2.2912
**A_24_P270144**	NM_001040034	CD63	2.2904
**A_23_P29594**	NM_052969	RPL39L	2.2894
**A_23_P68007**	NM_001679	ATP1B3	2.2868
**A_33_P3421913**	NM_014333	CADM1	2.2861
**A_24_P72479**	NM_006409	ARPC1A	2.2857
**A_23_P111311**	NM_144497	AKAP12	2.2856
**A_23_P57379**	NM_003504	CDC45	2.2825
**A_32_P134968**	NM_001024858	SPTB	2.2818
**A_23_P134714**	NM_005836	HRSP12	2.2818
**A_23_P388146**		ZNF587	2.2794
**A_23_P63660**	NM_032333	C10orf58	2.2782
**A_23_P104762**	NM_006106	YAP1	2.2751
**A_24_P192994**	NM_013402	FADS1	2.2742
**A_23_P301925**		COX1	2.2736
**A_23_P5903**	NM_016354	SLCO4A1	2.2728
**A_24_P108311**	NM_015277	NEDD4L	2.2712
**A_24_P319715**	NM_005742	PDIA6	2.2697
**A_23_P61487**	NM_018205	LRRC20	2.2693
**A_23_P20823**	NM_052844	WDR34	2.2687
**A_33_P3390357**	NM_001128626	SPIRE1	2.2682
**A_23_P137035**	NM_003662	PIR	2.2671
**A_23_P25674**	NM_001823	CKB	2.2664
**A_33_P3258612**	NM_002592	PCNA	2.2657
**A_33_P3313401**	NM_018947	CYCS	2.2651
**A_33_P3613516**		LOC254057	2.2628
**A_23_P216630**	NM_080546	SLC44A1	2.2618
**A_33_P3346806**		LOC100131581	2.2607
**A_33_P3323298**	NM_002228	JUN	2.2601
**A_23_P434900**	NM_144570	HN1L	2.2573
**A_23_P46369**	NM_002870	RAB13	2.2543
**A_24_P192727**		KAZALD1	2.2534
**A_23_P120056**	NM_033046	RTKN	2.2531
**A_23_P436284**	NM_178859	OSTBETA	2.2519
**A_32_P25050**	NM_172037	RDH10	2.2508
**A_23_P68851**	NM_001039570	KREMEN1	2.2504
**A_23_P19291**	NM_001069	TUBB2A	2.2462
**A_24_P149124**	NM_004772	C5orf13	2.2459
**A_23_P202658**	NM_000852	GSTP1	2.2453
**A_23_P88331**	NM_014750	DLGAP5	2.2424
**A_33_P3392077**	NM_004881	TP53I3	2.2417
**A_32_P190303**	NM_198461	LONRF2	2.2415
**A_24_P193295**	NM_198686	RAB15	2.2411

Online supplements are not copyedited prior to posting and the author(s) take full responsibility for the accuracy of all data.

**A_23_P57709**	NM_013363	PCOLCE2	2.2407
**A_23_P167040**	NM_006810	PDIA5	2.2385
**A_32_P186474**	NM_013277	RACGAP1	2.2379
**A_23_P216556**	NM_018424	EPB41L4B	2.2377
**A_33_P3409392**	NM_003506	FZD6	2.2357
**A_32_P135348**	NM_033394	TANC1	2.2292
**A_23_P29803**	NM_006232	POLR2H	2.2289
**A_23_P141345**	NM_001932	MPP3	2.2284
**A_32_P57702**	NR_033245	LOC641746	2.2283
**A_23_P56746**	NM_004460	FAP	2.2265
**A_23_P60028**	NM_005648	TCEB1	2.2264
**A_33_P3230548**	NM_014875	KIF14	2.2258
**A_24_P246351**	NM_138411	FAM71E1	2.2251
**A_24_P108451**	NM_000175	GPI	2.2250
**A_33_P3293573**	NM_001077621	VPS37D	2.2237
**A_23_P5441**	NM_005689	ABCB6	2.2223
**A_23_P38167**	NM_022036	GPRC5C	2.2213
**A_23_P102364**	NM_019850	NGEF	2.2209
**A_23_P114670**	NM_014448	ARHGEF16	2.2205
**A_23_P154806**	NM_012156	EPB41L1	2.2199
**A_33_P3226600**	NM_003801	GPAA1	2.2195
**A_32_P111996**	NR_038377	MGC39584	2.2182
**A_24_P204244**	NR_001562	ANXA2P1	2.2181
**A_24_P183094**	NM_024524	ATP13A3	2.2150
**A_23_P82478**	NM_019042	PUS7	2.2134
**A_23_P253301**	NM_053024	PFN2	2.2122
**A_23_P113005**	NM_004428	EFNA1	2.2108
**A_24_P48403**	NM_005433	YES1	2.2106
**A_23_P42080**	NM_014051	TMEM14A	2.2104
**A_33_P3230269**	NM_198182	GRHL1	2.2101
**A_23_P18196**	NM_002916	RFC4	2.2090
**A_23_P251421**	NM_031942	CDCA7	2.2084
**A_32_P132438**	NM_003488	AKAP1	2.2075
**A_23_P92441**	NM_002358	MAD2L1	2.2075
**A_33_P3313796**	NM_030771	CCDC34	2.2073
**A_23_P202810**	NM_004561	OVOL1	2.2071
**A_23_P62807**	NM_016002	SCCPDH	2.2068
**A_23_P42695**	NM_024051	GGCT	2.2050
**A_24_P388433**	NM_002718	PPP2R3A	2.2050
**A_23_P42664**	NM_006304	SHFM1	2.2040
**A_24_P334130**	NM_054034	FN1	2.2034
**A_24_P154006**	NM_005005	NDUFB9	2.2026
**A_23_P28953**	NM_175850	DNMT3B	2.1990
**A_23_P2223**	NM_002475	MYL6B	2.1927
**A_24_P100517**	NM_178448	C9orf140	2.1905
**A_33_P3402329**	NR_024607	MGC16121	2.1901

Online supplements are not copyedited prior to posting and the author(s) take full responsibility for the accuracy of all data.

**A_33_P3214481**	NM_001142595	P4HA1	2.1900
**A_23_P41804**	NM_033120	NKD2	2.1896
**A_23_P35219**	NM_002497	NEK2	2.1886
**A_23_P134953**	NM_001122	PLIN2	2.1884
**A_23_P365817**	NM_138689	PPP1R14B	2.1882
**A_23_P379034**	NM_025045	BAIAP2L2	2.1879
**A_23_P150510**	NM_153450	MED19	2.1873
**A_23_P330461**	NM_144686	TMC4	2.1867
**A_24_P179903**	NM_001625	AK2	2.1862
**A_32_P185317**	NR_038835	LOC645249	2.1853
**A_23_P210690**	NM_021158	TRIB3	2.1853
**A_23_P424561**	NM_133639	RHOV	2.1846
**A_23_P108437**	NM_003468	FZD5	2.1838
**A_24_P44462**	NM_000366	TPM1	2.1827
**A_24_P305764**	NM_004595	SMS	2.1826
**A_23_P124417**	NM_004336	BUB1	2.1810
**A_32_P215938**	NM_001145638	GPSM1	2.1790
**A_23_P82503**	NM_001040152	PEG10	2.1788
**A_23_P125829**	NM_000291	PGK1	2.1769
**A_23_P108294**	NM_177543	PPAP2C	2.1754
**A_23_P118246**	NM_016095	GINS2	2.1752
**A_32_P231568**	NM_152573	RASEF	2.1740
**A_23_P45365**	NM_033380	COL4A5	2.1722
**A_24_P319364**	NM_016946	F11R	2.1719
**A_23_P149206**	NM_003780	B4GALT2	2.1716
**A_23_P316850**	NM_182577	ODF3L2	2.1704
**A_23_P156739**	NM_032340	C6orf125	2.1689
**A_33_P3240747**	NR_039988	LOC100130899	2.1685
**A_33_P3341239**	NM_006201	CDK16	2.1668
**A_33_P3374210**	NM_002417	MKI67	2.1661
**A_23_P52336**	NM_170744	UNC5B	2.1657
**A_33_P3243153**	NM_002056	GFPT1	2.1632
**A_23_P215790**	NM_005228	EGFR	2.1626
**A_33_P3390102**	NM_005716	GIPC1	2.1626
**A_33_P3364348**	NM_020647	JPH1	2.1603
**A_23_P121602**	NM_003864	SAP30	2.1599
**A_23_P51085**	NM_020675	SPC25	2.1596
**A_23_P52806**	NM_012104	BACE1	2.1595
**A_23_P361049**	NM_012223	MYO1B	2.1574
**A_33_P3293213**	NM_001161616	RGL3	2.1550
**A_23_P141405**	NM_002512	NME2	2.1540
**A_23_P63402**	NM_013296	GPSM2	2.1536
**A_33_P3224105**	NM_001142761	C15orf23	2.1529
**A_23_P47885**	NM_153377	LRIG3	2.1511
**A_23_P80032**	NM_005225	E2F1	2.1496
**A_24_P48057**	NM_005853	IRX5	2.1475

Online supplements are not copyedited prior to posting and the author(s) take full responsibility for the accuracy of all data.

**A_33_P3236392**	NM_030916	PVRL4	2.1449
**A_32_P95729**	NM_018193	FANCI	2.1436
**A_33_P3405444**	NM_003941	WASL	2.1423
**A_24_P65507**	NM_144998	STRA13	2.1413
**A_23_P70231**	NM_001182	ALDH7A1	2.1403
**A_23_P393051**	NM_152365	C1orf172	2.1388
**A_33_P3397150**	NM_001190467	FLJ22184	2.1312
**A_23_P44363**	NM_020753	CASKIN2	2.1308
**A_23_P5983**	NM_006227	PLTP	2.1306
**A_23_P157809**	NM_012212	PTGR1	2.1299
**A_23_P124122**	NM_018663	PXMP2	2.1285
**A_33_P3214129**		LOC728061	2.1283
**A_33_P3216714**	NM_014787	DNAJC6	2.1268
**A_23_P29655**	NM_020685	C3orf14	2.1266
**A_33_P3335590**	NM_001103175	CCDC64B	2.1250
**A_23_P319859**	NM_005244	EYA2	2.1242
**A_23_P201628**	NM_002293	LAMC1	2.1234
**A_33_P3386547**	NM_152386	SGPP2	2.1198
**A_23_P88731**	NM_002875	RAD51	2.1196
**A_33_P3352557**	NM_032110	DMRTA2	2.1183
**A_23_P88522**	NM_021077	NMB	2.1174
**A_23_P54840**	NM_005946	MT1A	2.1168
**A_23_P16078**	NM_002573	PAFAH1B3	2.1154
**A_24_P413884**	NM_001809	CENPA	2.1142
**A_33_P3214466**	NM_018670	MESP1	2.1132
**A_24_P182122**		ND1	2.1124
**A_23_P161297**	NM_018245	OGDHL	2.1117
**A_23_P164814**	NM_024323	C19orf57	2.1115
**A_23_P12463**	NM_002826	QSOX1	2.1112
**A_23_P200507**	NM_014184	CNIH4	2.1101
**A_24_P38895**	NM_002105	H2AFX	2.1083
**A_23_P49448**	NM_024306	FA2H	2.1079
**A_23_P103511**	NM_001085375	C1orf226	2.1057
**A_23_P421306**	NM_177963	SYT12	2.1056
**A_33_P3243337**	NM_019107	C19orf10	2.1055
**A_32_P10133**	NR_023388	PRINS	2.1053
**A_23_P211909**	NM_002670	PLS1	2.1045
**A_23_P66117**	NM_032039	ITFG3	2.1029
**A_33_P3254606**	NM_001083961	WDR62	2.1022
**A_33_P3314579**	NM_001142625	RAB34	2.1018
**A_23_P44684**	NM_018098	ECT2	2.1014
**A_24_P365807**	NM_004429	EFNB1	2.1011
**A_24_P57367**	NM_000687	AHCY	2.0975
**A_33_P3336696**		ND4L	2.0960
**A_23_P210210**	NM_001430	EPAS1	2.0950
**A_23_P128147**	NM_006082	TUBA1B	2.0942

Online supplements are not copyedited prior to posting and the author(s) take full responsibility for the accuracy of all data.

**A_24_P77364**	NM_212552	BOLA3	2.0941
**A_33_P3347040**	NM_001242901	LOC100131094	2.0939
**A_33_P3372074**	NM_001031803	LLGL2	2.0916
**A_23_P81770**	NM_003463	PTP4A1	2.0912
**A_19_P00317447**		XLOC_002736	2.0905
**A_23_P428842**	NM_138399	TMEM44	2.0901
**A_23_P431853**		ND2	2.0899
**A_24_P193435**	NM_003257	TJP1	2.0887
**A_24_P181585**	NM_018509	LRRC59	2.0884
**A_23_P218331**	NM_001017916	CYB561	2.0883
**A_23_P333951**	NM_144989	DNAH14	2.0874
**A_23_P17870**	NM_012264	TMEM184B	2.0869
**A_23_P143935**	NM_025163	PIGZ	2.0865
**A_33_P3716128**	NM_005496	SMC4	2.0863
**A_23_P128728**	NM_001172	ARG2	2.0858
**A_33_P3270657**	NM_198947	FAM111B	2.0858
**A_23_P133236**	NM_018934	PCDHB14	2.0829
**A_23_P120316**	NM_006636	MTHFD2	2.0808
**A_23_P153676**	NM_003260	TLE2	2.0806
**A_23_P8900**	NM_004374	COX6C	2.0805
**A_24_P314571**		SPC24	2.0803
**A_23_P253896**	NM_001033047	NPNT	2.0800
**A_33_P3216008**	NM_145061	SKA3	2.0796
**A_23_P18579**	NM_006607	PTTG2	2.0795
**A_23_P141730**	NM_001943	DSG2	2.0793
**A_24_P813147**	NM_177987	TUBB8	2.0790
**A_32_P103695**	NM_145269	FAM92A1	2.0788
**A_23_P140434**	NM_018728	MYO5C	2.0779
**A_23_P256956**	NM_005733	KIF20A	2.0768
**A_23_P153026**	NM_000152	GAA	2.0758
**A_23_P121657**	NM_005114	HS3ST1	2.0754
**A_23_P139547**	NM_006009	TUBA1A	2.0753
**A_24_P35228**	NM_024915	GRHL2	2.0741
**A_24_P239731**	NM_004776	B4GALT5	2.0728
**A_33_P3405728**	NM_004572	PKP2	2.0699
**A_23_P22096**	NM_153831	PTK2	2.0692
**A_24_P398147**	NM_006393	NEBL	2.0687
**A_23_P147786**	NM_014677	RIMS2	2.0680
**A_32_P202703**	NM_001242480	LOC389831	2.0673
**A_23_P114405**	NM_012286	MORF4L2	2.0670
**A_23_P318300**	NM_133646	ZAK	2.0655
**A_23_P53276**	NM_003920	TIMELESS	2.0654
**A_23_P68717**	NM_003720	PSMG1	2.0641
**A_24_P4054**	NM_003302	TRIP6	2.0629
**A_33_P3271121**		SH3BP4	2.0624
**A_23_P49865**	NM_015982	YBX2	2.0621

Online supplements are not copyedited prior to posting and the author(s) take full responsibility for the accuracy of all data.

**A_33_P3263666**	NM_152326	ANKRD9	2.0612
**A_24_P945283**	NM_021120	DLG3	2.0607
**A_23_P399501**	NM_182470	PKM2	2.0601
**A_23_P152235**	NM_024336	IRX3	2.0599
**A_33_P3318796**	NM_005860	FSTL3	2.0582
**A_23_P48166**	NM_002822	TWF1	2.0578
**A_23_P128991**	NM_031210	SLIRP	2.0574
**A_33_P3278649**	NM_203468	ENTPD2	2.0571
**A_23_P403335**	NM_015065	EXPH5	2.0562
**A_33_P3408320**	NM_198207	CERS1	2.0553
**A_33_P3273020**	NM_004470	FKBP2	2.0546
**A_23_P382188**	NM_001013841	STAP2	2.0546
**A_23_P201342**	NM_004421	DVL1	2.0544
**A_33_P3209229**	NM_014353	RAB26	2.0522
**A_23_P253350**	NM_020130	C8orf4	2.0515
**A_23_P306215**	NM_145175	FAM84A	2.0514
**A_23_P57588**	NM_016426	GTSE1	2.0497
**A_23_P157795**	NM_003798	CTNNAL1	2.0457
**A_23_P356494**	NM_006846	SPINK5	2.0443
**A_23_P23303**	NM_003686	EXO1	2.0436
**A_32_P328023**	NM_018383	WDR33	2.0434
**A_23_P380766**	NM_006035	CDC42BPB	2.0430
**A_32_P218355**	NM_001164446	C6orf132	2.0426
**A_23_P164826**	NM_006397	RNASEH2A	2.0421
**A_23_P142849**	NM_005168	RND3	2.0404
**A_23_P315933**	NM_148912	ABHD11	2.0404
**A_23_P258321**	NM_015969	MRPS17	2.0403
**A_23_P434890**	NM_014550	CARD10	2.0400
**A_33_P3271241**	NM_021129	PPA1	2.0397
**A_23_P135499**	NM_013943	CLIC4	2.0391
**A_23_P100315**	NM_020664	DECR2	2.0363
**A_33_P3335725**	NM_000208	INSR	2.0362
**A_23_P50000**	NM_024792	FAM57A	2.0360
**A_23_P163682**	NM_022450	RHBDF1	2.0360
**A_23_P25735**	NM_002791	PSMA6	2.0339
**A_33_P3691916**	NM_014883	FAM13A	2.0337
**A_33_P3265185**		CSNK1E	2.0322
**A_33_P3396607**	NM_003359	UGDH	2.0315
**A_23_P253221**	NM_032995	ARHGEF4	2.0313
**A_33_P3263538**		NEAT1	2.0300
**A_33_P3387050**	NM_001001795	C8orf82	2.0294
**A_23_P101642**	NM_002842	PTPRH	2.0294
**A_23_P418373**	NM_004050	BCL2L2	2.0282
**A_24_P191664**	NM_014498	GOLIM4	2.0271
**A_24_P835500**	NM_001034841	ITPRIPL2	2.0231
**A_23_P90659**	NM_014713	LAPTM4A	2.0219

Online supplements are not copyedited prior to posting and the author(s) take full responsibility for the accuracy of all data.

**A_23_P416468**	NM_025049	PIF1	2.0208
**A_24_P315014**	XR_113307	XAGE-4	2.0199
**A_24_P173823**	NM_002585	PBX1	2.0190
**A_33_P3373469**	NM_002808	PSMD2	2.0187
**A_23_P108404**	NM_001037131	AGAP1	2.0181
**A_23_P16469**	NM_001005377	PLAUR	2.0177
**A_23_P259586**	NM_003318	TTK	2.0176
**A_23_P369701**	NM_021214	FAM108C1	2.0160
**A_33_P3278573**	NM_001099680	MAGIX	2.0145
**A_33_P3349536**	NM_001114121	CHEK1	2.0105
**A_23_P82748**	NM_020189	ENY2	2.0099
**A_24_P391526**	NM_001005333	MAGED1	2.0094
**A_23_P203900**	NM_005505	SCARB1	2.0085
**A_23_P8906**	NM_013437	LRP12	2.0082
**A_23_P146644**	NM_001002857	ANXA2	2.0075
**A_33_P3311076**	NM_001190807	CYB5A	2.0068
**A_23_P32707**	NM_012291	ESPL1	2.0065
**A_32_P168247**	NM_004373	COX6A1	2.0053
**A_23_P123974**	NM_012145	DTYMK	2.0051
**A_24_P329487**	NM_174911	FAM84B	2.0016
**A_23_P9603**	NM_006904	PRKDC	2.0015
**A_24_P944253**	NM_130446	KLHL6	-2.0004
**A_24_P288836**	NR_001435	HLA-DPB2	-2.0026
**A_23_P357811**	NM_021038	MBNL1	-2.0032
**A_24_P305345**	NM_021155	CD209	-2.0060
**A_33_P3364808**	NM_002838	PTPRC	-2.0071
**A_33_P3332970**	NM_005127	CLEC2B	-2.0097
**A_33_P3351536**	NM_173174	PTK2B	-2.0112
**A_33_P3222942**	NM_080819	GPR78	-2.0146
**A_33_P3288844**	NM_000565	IL6R	-2.0147
**A_33_P3323760**	NR_015447	LOC153684	-2.0165
**A_23_P52647**	NM_006795	EHD1	-2.0172
**A_23_P129466**	NM_024997	ATF7IP2	-2.0179
**A_24_P242036**	NR_002184	RRP7B	-2.0202
**A_23_P78053**	NM_030802	FAM117A	-2.0219
**A_23_P156683**	NM_000595	LTA	-2.0223
**A_23_P12082**	NM_001025199	CHI3L2	-2.0224
**A_23_P166087**	NM_014737	RASSF2	-2.0228
**A_23_P165333**	NM_139346	BIN1	-2.0236
**A_23_P151075**	NM_001175	ARHGDIB	-2.0239
**A_23_P50678**	NM_139355	MATK	-2.0261
**A_33_P3221019**	NM_173059	ZAN	-2.0262
**A_24_P263910**		C14orf182	-2.0280
**A_23_P412562**	NM_174896	C1orf162	-2.0291
**A_23_P162486**	NM_002831	PTPN6	-2.0313
**A_33_P3214072**	XM_001129558	LOC729175	-2.0325

Online supplements are not copyedited prior to posting and the author(s) take full responsibility for the accuracy of all data.

**A_24_P396167**	NM_001335	CTSW	-2.0332
**A_23_P14564**	NM_003608	GPR65	-2.0335
**A_32_P703**	XM_942822	LOC646626	-2.0349
**A_33_P3279362**	NR_027021	GRIK1-AS1	-2.0372
**A_33_P3273474**	NM_001765	CD1C	-2.0375
**A_23_P152838**	NM_002985	CCL5	-2.0386
**A_23_P34744**	NM_000396	CTSK	-2.0424
**A_24_P64344**	NM_013314	BLNK	-2.0454
**A_23_P27606**	NM_004843	IL27RA	-2.0457
**A_33_P3367565**		FNBP1	-2.0478
**A_33_P3341970**	NM_173808	NEGR1	-2.0528
**A_33_P3382324**	NM_012217	TPSD1	-2.0533
**A_23_P35564**	NM_015490	SEC31B	-2.0545
**A_32_P169179**	NR_002307	MSX2P1	-2.0579
**A_24_P379104**	NM_006875	PIM2	-2.0621
**A_33_P3371650**		STK17B	-2.0631
**A_23_P155257**	NM_032682	FOXP1	-2.0641
**A_23_P342131**	NM_153611	CYBASC3	-2.0660
**A_23_P147025**	NM_004794	RAB33A	-2.0674
**A_33_P3447441**		LOC202025	-2.0676
**A_33_P3377190**		LOC100131662	-2.0686
**A_24_P71244**	NM_005026	PIK3CD	-2.0696
**A_33_P3387155**	NR_002814	LOC374443	-2.0698
**A_33_P3254335**	NM_207406	BEND4	-2.0729
**A_33_P3414202**	NM_001170704	MBNL3	-2.0738
**A_24_P329065**	NM_007048	BTN3A1	-2.0772
**A_23_P125618**	NM_000808	GABRA3	-2.0782
**A_23_P329112**	NM_000215	JAK3	-2.0782
**A_23_P11070**	NM_032553	GPR174	-2.0796
**A_33_P3330952**	NM_006095	ATP8A1	-2.0805
**A_19_P00809368**		XLOC_002749	-2.0850
**A_33_P3352827**	NM_003037	SLAMF1	-2.0864
**A_23_P74278**	NM_001037341	PDE4B	-2.0885
**A_33_P3227880**	NM_001197247	BTN3A2	-2.0898
**A_23_P22660**	NM_006639	CYSLTR1	-2.0909
**A_23_P364792**	NM_001005852	CYorf15A	-2.0911
**A_23_P117662**	NM_002112	HDC	-2.0916
**A_23_P148473**	NM_000206	IL2RG	-2.0970
**A_24_P278747**	NM_001759	CCND2	-2.0974
**A_33_P3212232**	NM_001039396	MPEG1	-2.0988
**A_23_P155688**	NM_021114	SPINK2	-2.1009
**A_33_P3436646**		LOC151657	-2.1037
**A_33_P3290443**	NR_002569	SCARNA9	-2.1042
**A_24_P366122**	NM_024722	ACBD4	-2.1048
**A_24_P386746**	NM_031491	RBP5	-2.1050
**A_33_P3360341**	NM_001002295	GATA3	-2.1083

Online supplements are not copyedited prior to posting and the author(s) take full responsibility for the accuracy of all data.

**A_33_P3220919**	NM_005160	ADRBK2	-2.1092
**A_23_P305092**	NM_019604	CRTAM	-2.1099
**A_23_P170574**	NM_178310	SNAI3	-2.1123
**A_23_P257815**	NM_005582	CD180	-2.1127
**A_32_P46214**	NM_173653	SLC9A9	-2.1211
**A_23_P151805**	NM_006329	FBLN5	-2.1228
**A_33_P3303857**	NM_001184714	SLAMF6	-2.1231
**A_24_P68079**	NM_014831	TRANK1	-2.1254
**A_24_P97374**	NM_005442	EOMES	-2.1255
**A_33_P3220723**	NM_015196	KIAA0922	-2.1278
**A_23_P27424**	NM_133460	ZNF418	-2.1288
**A_33_P3368139**	NM_005921	MAP3K1	-2.1322
**A_33_P3226995**	NM_006137	CD7	-2.1342
**A_32_P815507**		LOC100130920	-2.1343
**A_23_P134851**	NM_003974	DOK2	-2.1348
**A_23_P258164**	NM_001302	CORT	-2.1356
**A_23_P501722**	NM_139022	TSPAN32	-2.1372
**A_23_P201731**	NM_004619	TRAF5	-2.1406
**A_24_P139901**	NM_002101	GYPC	-2.1436
**A_33_P3415032**	NM_002232	KCNA3	-2.1457
**A_24_P299911**	NM_015148	PASK	-2.1466
**A_23_P214360**	NM_002460	IRF4	-2.1520
**A_24_P172481**	NM_006074	TRIM22	-2.1565
**A_23_P73429**	NM_005335	HCLS1	-2.1598
**A_24_P355649**	NM_002017	FLI1	-2.1635
**A_33_P3411477**	NM_001001414	NCCRP1	-2.1645
**A_33_P3367171**		SLC22A8	-2.1666
**A_23_P44207**	NM_130767	ACOT12	-2.1764
**A_24_P336584**	NM_182664	RASSF5	-2.1804
**A_23_P438**	NM_017773	LAX1	-2.1808
**A_23_P87742**	NM_001039670	IFFO1	-2.1815
**A_24_P384397**	NM_133452	RAVER1	-2.1837
**A_33_P3262515**	NM_007368	RASA3	-2.1838
**A_23_P37702**	NM_003294	TPSAB1	-2.1842
**A_24_P206343**	NM_033054	MYO1G	-2.1853
**A_23_P70670**	NM_004233	CD83	-2.1891
**A_32_P56249**	NR_038996	LOC100131733	-2.1904
**A_33_P3268564**	NM_001004722	NCK2	-2.1962
**A_23_P112452**	NR_003191	GGTA1P	-2.1972
**A_23_P259741**	NM_002971	SATB1	-2.2006
**A_33_P3242614**	NR_002454	LOC606724	-2.2018
**A_33_P3212092**	NM_145341	PDCD4	-2.2023
**A_23_P416747**	NM_000733	CD3E	-2.2028
**A_33_P3413993**		SERPING1	-2.2176
**A_23_P200741**	NM_001937	DPT	-2.2197
**A_33_P3237927**	NM_001164741	ARHGAP4	-2.2215

Online supplements are not copyedited prior to posting and the author(s) take full responsibility for the accuracy of all data.

**A_32_P176911**	NR_026774	NCRNA00239	-2.2224
**A_24_P65941**	NR_026812	C21orf96	-2.2265
**A_23_P78742**	NM_001459	FLT3LG	-2.2335
**A_23_P154962**	NM_015672	RIMBP3	-2.2355
**A_23_P209726**	NM_007237	SP140	-2.2358
**A_23_P250212**	NM_001080826	SGK223	-2.2364
**A_24_P45476**	NM_002995	XCL1	-2.2383
**A_23_P1374**	NM_006257	PRKCQ	-2.2393
**A_23_P38959**	NM_005428	VAV1	-2.2397
**A_23_P66694**	NM_006495	EVI2B	-2.2429
**A_23_P219060**	NM_022107	GPSM3	-2.2432
**A_23_P420281**	NM_002738	PRKCB	-2.2444
**A_23_P408285**	NM_153026	PRICKLE1	-2.2444
**A_23_P207911**	NM_016113	TRPV2	-2.2486
**A_33_P3337009**		PPCS	-2.2493
**A_33_P3304170**	NM_002649	PIK3CG	-2.2539
**A_33_P3215640**	NM_153370	PI16	-2.2545
**A_23_P13382**	NM_001013254	LSP1	-2.2551
**A_33_P3216448**	NM_001163771	COL11A2	-2.2560
**A_33_P3251771**	NM_015247	CYLD	-2.2589
**A_23_P141555**	NM_013351	TBX21	-2.2598
**A_33_P3274134**	NM_001137560	TMEM151B	-2.2605
**A_23_P205370**	NM_016150	ASB2	-2.2606
**A_33_P3224878**	NM_000885	ITGA4	-2.2618
**A_33_P3259865**	NR_033186	C1orf220	-2.2622
**A_19_P00315528**		XLOC_008370	-2.2636
**A_33_P3313929**	NM_031409	CCR6	-2.2650
**A_23_P376060**	NM_012481	IKZF3	-2.2684
**A_24_P751074**	NM_005238	ETS1	-2.2690
**A_33_P3229869**	NR_040082	LOC100128714	-2.2755
**A_33_P3235213**	NM_173799	TIGIT	-2.2765
**A_23_P37736**	NM_001192	TNFRSF17	-2.2765
**A_33_P3391275**	NR_026958	LOC284749	-2.2769
**A_23_P250413**	NM_022141	PARVG	-2.2789
**A_24_P865**	NM_206921	C6orf204	-2.2842
**A_33_P3420446**	NM_001161528	LRRD1	-2.2878
**A_32_P179998**	NM_033053	DMRTC1	-2.2945
**A_33_P3391796**	NM_005450	NOG	-2.2950
**A_23_P118095**	NM_005061	RPL3L	-2.3009
**A_33_P3294986**	NM_005357	LIPE	-2.3072
**A_33_P3318414**	NM_012292	HMHA1	-2.3084
**A_23_P128808**	NM_013345	GPR132	-2.3088
**A_33_P3243554**	NM_145802	38961.00	-2.3125
**A_33_P3327265**	NM_001098815	KIAA0748	-2.3149
**A_23_P90626**	NM_004288	CYTIP	-2.3222
**A_23_P402319**	NM_152687	GAPT	-2.3331

Online supplements are not copyedited prior to posting and the author(s) take full responsibility for the accuracy of all data.

**A_23_P78092**	NM_001003927	EVI2A	-2.3341
**A_23_P53763**	NM_025113	C13orf18	-2.3381
**A_33_P3389153**	NM_005990	STK10	-2.3384
**A_33_P3364308**		OTOA	-2.3414
**A_23_P205567**	NM_006255	PRKCH	-2.3426
**A_33_P3286684**		IKZF1	-2.3510
**A_33_P3350259**	NM_001098524	FAM129C	-2.3530
**A_33_P3236734**	NM_001204118	CLEC17A	-2.3552
**A_23_P502142**	NM_002037	FYN	-2.3588
**A_33_P3359900**	NM_198449	EMB	-2.3616
**A_23_P321984**	NM_172004	CLECL1	-2.3627
**A_23_P76529**	NM_000889	ITGB7	-2.3631
**A_23_P376488**	NM_000594	TNF	-2.3665
**A_33_P3387991**	NM_001805	CEBPE	-2.3689
**A_23_P137238**	NM_004653	KDM5D	-2.3697
**A_23_P142974**	NM_001007231	ARHGAP25	-2.3763
**A_33_P3279720**		ARFRP1	-2.3784
**A_23_P46356**	NM_024575	TNFAIP8L2	-2.3802
**A_23_P16722**	NM_014689	DOCK10	-2.3898
**A_23_P107336**	NM_014716	ACAP1	-2.3928
**A_33_P3214625**	NM_003866	INPP4B	-2.3964
**A_23_P51231**	NM_001031680	RUNX3	-2.3972
**A_23_P305198**	NM_003151	STAT4	-2.4042
**A_33_P3303697**	NM_001006658	CR2	-2.4115
**A_23_P357104**	NM_001155	ANXA6	-2.4121
**A_24_P89891**	NM_005658	TRAF1	-2.4160
**A_23_P15146**	NM_001012631	IL32	-2.4178
**A_23_P96590**	NM_014710	GPRASP1	-2.4375
**A_23_P124642**	NM_005739	RASGRP1	-2.4429
**A_23_P106675**	NM_002661	PLCG2	-2.4450
**A_24_P203000**	NM_000878	IL2RB	-2.4482
**A_33_P3645465**	NR_026932	LOC282997	-2.4491
**A_23_P10025**	NM_006159	NELL2	-2.4501
**A_23_P500861**	NM_182961	SYNE1	-2.4512
**A_33_P3260614**	NM_004573	PLCB2	-2.4520
**A_33_P3350094**	NM_001145112	PATL2	-2.4579
**A_23_P302018**	NM_003328	TXK	-2.4580
**A_23_P41365**	NM_012390	SMR3A	-2.4588
**A_24_P290751**	NM_004416	DTX1	-2.4607
**A_23_P368681**	NM_015660	GIMAP2	-2.4701
**A_23_P22444**	NM_002621	CFP	-2.4800
**A_23_P145631**	NM_024711	GIMAP6	-2.4820
**A_32_P219520**	NM_014350	TNFAIP8	-2.4878
**A_33_P3293918**	NM_170600	SH2D3C	-2.4885
**A_24_P209455**	NM_018326	GIMAP4	-2.4966
**A_19_P00322333**		XLOC_012197	-2.4966

Online supplements are not copyedited prior to posting and the author(s) take full responsibility for the accuracy of all data.

**A_33_P3368830**	NM_001033667	LY9	-2.5049
**A_32_P48825**	NM_080747	KRT72	-2.5070
**A_33_P3212172**	NM_024798	SNX22	-2.5130
**A_23_P137046**	NM_022567	NYX	-2.5342
**A_23_P48997**	NM_003978	PSTPIP1	-2.5415
**A_33_P3257993**	NM_017831	RNF125	-2.5470
**A_23_P126278**	NM_003465	CHIT1	-2.5533
**A_32_P453321**	NM_001145636	C1orf228	-2.5595
**A_23_P118025**	NM_022355	DPEP2	-2.5604
**A_23_P100963**	NM_182538	SPNS3	-2.5645
**A_23_P315571**	NM_015150	RFTN1	-2.5651
**A_33_P3212799**	NR_026997	C22orf34	-2.5709
**A_24_P393740**	NM_001465	FYB	-2.5728
**A_33_P3329433**		TTN	-2.5817
**A_33_P3253687**	NR_003945	GVINP1	-2.5853
**A_23_P98350**	NM_001165	BIRC3	-2.5911
**A_23_P106761**	NM_007074	CORO1A	-2.6017
**A_24_P295010**	NM_004155	SERPINB9	-2.6041
**A_33_P3213235**	NM_198492	CLEC4G	-2.6124
**A_23_P203173**	NM_001558	IL10RA	-2.6161
**A_23_P164691**	NM_002162	ICAM3	-2.6195
**A_32_P145010**		LOC729683	-2.6216
**A_23_P354805**	NM_007249	KLF12	-2.6242
**A_23_P98910**	NM_006152	LRMP	-2.6256
**A_33_P3400273**	NM_000655	SELL	-2.6273
**A_23_P64661**	NM_032496	ARHGAP9	-2.6347
**A_24_P227927**	NM_181078	IL21R	-2.6354
**A_23_P320739**	NM_002397	MEF2C	-2.6356
**A_23_P132515**	NM_017699	SIDT1	-2.6370
**A_33_P3415698**	NM_144615	TMIGD2	-2.6436
**A_23_P5002**	NM_001042600	MAP4K1	-2.6504
**A_33_P3380383**	NM_001099221	TIFAB	-2.6592
**A_23_P107735**	NM_001783	CD79A	-2.6595
**A_33_P3316544**	NM_001113523	PARP15	-2.6651
**A_23_P140427**	NM_016337	EVL	-2.6688
**A_23_P23279**	NM_052862	RCSD1	-2.6888
**A_23_P25566**	NM_004951	GPR183	-2.6920
**A_24_P276576**	NM_032738	FCRLA	-2.6955
**A_23_P126584**	NM_005894	CD5L	-2.7013
**A_23_P14302**	NR_026779	C14orf139	-2.7036
**A_33_P3280950**	NR_026971	LOC144571	-2.7072
**A_23_P39386**	NM_014266	HCST	-2.7225
**A_23_P500741**	NM_005187	CBFA2T3	-2.7233
**A_23_P162607**	NM_017564	STAB2	-2.7259
**A_23_P85952**	NM_024901	DENND2D	-2.7333
**A_23_P103104**	NM_002405	MFNG	-2.7433

Online supplements are not copyedited prior to posting and the author(s) take full responsibility for the accuracy of all data.

**A_23_P160751**	NM_030764	FCRL2	-2.7440
**A_23_P433785**	NM_002561	P2RX5	-2.7482
**A_33_P3380462**	NM_022049	GPR88	-2.7599
**A_23_P152655**	NM_000873	ICAM2	-2.7714
**A_23_P84154**	NM_018460	ARHGAP15	-2.7881
**A_23_P119196**	NM_016270	KLF2	-2.7920
**A_33_P3336760**	NM_018849	ABCB4	-2.7937
**A_23_P84596**	NM_016459	MZB1	-2.8003
**A_24_P95723**	NR_026800	KIAA0125	-2.8032
**A_23_P70688**	NM_004271	LY86	-2.8108
**A_23_P206806**	NM_002209	ITGAL	-2.8291
**A_24_P153840**	NM_033086	FGD3	-2.8427
**A_23_P211561**	NM_152513	MEI1	-2.8441
**A_23_P26325**	NM_002987	CCL17	-2.8490
**A_24_P237443**	NM_018990	SASH3	-2.8503
**A_23_P360804**	NM_020939	CPNE5	-2.8550
**A_33_P3294177**		LOC100131043	-2.8614
**A_23_P49376**	NM_000078	CETP	-2.8659
**A_33_P3286157**	NM_003327	TNFRSF4	-2.8719
**A_33_P3241021**	NR_026672	CD69	-2.8819
**A_33_P3227443**	NM_175900	C16orf54	-2.8869
**A_33_P3414880**	XR_115108	LOC339192	-2.9025
**A_33_P3368014**	NM_001040107	HVCN1	-2.9105
**A_23_P153897**	NM_052847	GNG7	-2.9122
**A_23_P114299**	NM_001504	CXCR3	-2.9128
**A_33_P3343120**	NM_002163	IRF8	-2.9200
**A_23_P100730**	NM_003726	SKAP1	-2.9354
**A_23_P61149**	NM_001017915	INPP5D	-2.9365
**A_23_P414654**	NM_175738	RAB37	-2.9453
**A_23_P91764**	NM_052945	TNFRSF13C	-2.9485
**A_33_P3333960**	NR_024464	LOC100188949	-2.9630
**A_33_P3281273**	NM_003775	S1PR4	-2.9662
**A_23_P314250**	NM_033387	FAM78A	-2.9691
**A_23_P99442**	NM_004119	FLT3	-2.9754
**A_23_P81441**	NM_130848	C5orf20	-2.9843
**A_32_P37592**	NR_003003	SCARNA17	-3.0233
**A_24_P73599**	NM_172217	IL16	-3.0361
**A_23_P306941**	NM_153615	RGL4	-3.0520
**A_23_P404481**	NM_001400	S1PR1	-3.0636
**A_23_P209055**	NM_001771	CD22	-3.0707
**A_23_P201778**	NM_080588	PTPN7	-3.0715
**A_23_P156218**	NM_002104	GZMK	-3.0805
**A_23_P78608**	NM_024898	DENND1C	-3.0991
**A_23_P171074**	NM_004867	ITM2A	-3.1277
**A_32_P77102**	NR_038461	LOC100128420	-3.1340
**A_23_P310931**	NM_001841	CNR2	-3.1434

Online supplements are not copyedited prior to posting and the author(s) take full responsibility for the accuracy of all data.

**A_23_P427023**	NM_130759	GIMAP1	-3.1648
**A_33_P3358923**	NM_181780	BTLA	-3.1967
**A_23_P354151**	NM_005546	ITK	-3.2093
**A_33_P3383970**	NM_030956	TLR10	-3.2146
**A_33_P3462422**	NM_001164685	THEMIS	-3.2299
**A_23_P101683**	NM_001828	CLC	-3.2369
**A_23_P212568**	NM_016388	TRAT1	-3.2421
**A_33_P3234202**	NM_004944	DNASE1L3	-3.2453
**A_23_P44155**	NM_198196	CD96	-3.2572
**A_33_P3297345**	NM_001105669	TTC24	-3.2602
**A_33_P3398143**		ITGB2	-3.2643
**A_32_P101352**	NM_001025265	CXorf65	-3.2717
**A_32_P175934**	NM_001778	CD48	-3.2761
**A_23_P401076**	NM_145006	SUSD3	-3.2799
**A_23_P43369**	NM_014450	SIT1	-3.2872
**A_24_P82749**	NM_001774	CD37	-3.3077
**A_23_P253317**	NM_013308	GPR171	-3.3351
**A_24_P169234**	NM_001079	ZAP70	-3.3361
**A_33_P3299279**	NM_001014279	C5orf39	-3.3475
**A_32_P232559**	NR_036502	LOC439949	-3.3737
**A_23_P44112**	NM_014387	LAT	-3.3842
**A_33_P3382746**	NM_005356	LCK	-3.3902
**A_23_P28857**	NM_018556	SIRPG	-3.3961
**A_23_P30736**	NM_002120	HLA-DOB	-3.4037
**A_23_P371215**	NM_012092	ICOS	-3.4462
**A_23_P42588**	NM_018384	GIMAP5	-3.4559
**A_23_P79069**	NM_022904	RASAL3	-3.4562
**A_23_P21057**	NM_052838	37135.00	-3.4791
**A_33_P3341105**	NM_002602	PDE6G	-3.5127
**A_23_P58132**	NM_004310	RHOH	-3.5715
**A_23_P85800**	NM_001803	CD52	-3.5894
**A_23_P34676**	NM_198053	CD247	-3.6549
**A_33_P3250671**	NM_003202	TCF7	-3.6564
**A_33_P3282556**	NM_024600	TMEM204	-3.7041
**A_24_P274831**	NM_153236	GIMAP7	-3.7259
**A_23_P120902**	NM_006498	LGALS2	-3.7543
**A_33_P3377151**	NM_006274	CCL19	-3.7657
**A_23_P340019**	NM_178844	NLRC3	-3.7835
**A_24_P340128**	NM_178129	P2RY8	-3.8571
**A_23_P253321**	NM_006228	PNOC	-3.8819
**A_23_P17134**	NM_002371	MAL	-4.0131
**A_23_P98410**	NM_000073	CD3G	-4.0357
**A_33_P3328559**	NM_198517	TBC1D10C	-4.1133
**A_23_P167168**	NM_144646	IGJ	-4.1280
**A_32_P206479**	NM_178457	ZNF831	-4.1560
**A_23_P99275**	NM_002258	KLRB1	-4.1663

Online supplements are not copyedited prior to posting and the author(s) take full responsibility for the accuracy of all data.

**A_23_P14165**	NM_005292	GPR18	-4.2149
**A_23_P312920**	NM_006235	POU2AF1	-4.3070
**A_23_P218369**	NM_032963	CCL14	-4.3227
**A_23_P138125**	NM_005449	FAIM3	-4.3973
**A_23_P207201**	NM_001039933	CD79B	-4.4102
**A_33_P3375541**	NM_000732	CD3D	-4.5050
**A_24_P20630**	NM_016269	LEF1	-4.5123
**A_33_P3295056**	NM_005608	PTPRCAP	-4.5588
**A_33_P3351745**	NM_024070	PVRIG	-4.6996
**A_23_P311875**	NM_006725	CD6	-4.7002
**A_23_P10232**	NM_017935	BANK1	-4.8173
**A_23_P7503**	NM_138379	TIMD4	-4.8377
**A_32_P8813**	NR_024433	LOC283663	-4.8884
**A_23_P48088**	NM_001242	CD27	-4.9272
**A_23_P404494**	NM_002185	IL7R	-4.9333
**A_33_P3298990**	NM_014207	CD5	-5.0199
**A_33_P3248265**	NM_002341	LTB	-5.1891
**A_33_P3406567**	NM_152866	MS4A1	-5.5392
**A_24_P252945**	NM_032966	CXCR5	-5.5841
**A_23_P324384**	NM_001039567	RPS4Y2	-5.6035
**A_23_P39067**	NM_003121	SPIB	-5.8169
**A_23_P259314**	NM_001008	RPS4Y1	-6.1386
**A_23_P31725**	NM_001715	BLK	-6.6225
**A_23_P113572**	NM_001770	CD19	-6.7802
**A_33_P3299254**	NM_013378	VPREB3	-6.8046
**A_23_P140384**	NM_001911	CTSG	-6.9978
**A_33_P3273623**	NM_002989	CCL21	-7.0044
**A_33_P3250680**	NM_000074	CD40LG	-7.0226
**A_23_P343398**	NM_001838	CCR7	-7.2997
**A_23_P357717**	NM_021966	TCL1A	-8.3047

C=Cancer, R=Reactive.

Online supplements are not copyedited prior to posting and the author(s) take full responsibility for the accuracy of all data.

e-Table 6. Transcription factor enrichment of genes that show significantly higher expression in granulomatous lymph nodes.

Transcription factor	Z-score*
RELA	20.96
NF-kappaB	20.079
EBF1	18.258
ZNF354C	17.317
REL	16.476
MZF1_1-4	15.3
MZF1_5-13	14.712
Tcfcp2l1	14.683
NFKB1	14.039
FEV	13.884
ELF5	12.829
CTCF	12.714
SPI1	12.541
RUNX1	10.99
Stat3	10.639
Myf	10.557
HNF4A	10.397
SP1	10.125
Hand1::Tcfe2a	10.105
SPIB	10.009

*z-scores of >10 are considered to indicate highly significant over representation of transcription factor binding sites (TFBS) within the analysed gene list.

Online supplements are not copyedited prior to posting and the author(s) take full responsibility for the accuracy of all data.

e-Table 7. Transcription factor enrichment of genes that show significantly higher expression in malignant lymph nodes.

Transcription factor	Z-score
Klf4	38.732
Zfx	33.501
SP1	31.432
MZF1_5-13	24.617
Tcfcp2l1	22.421
ZNF354C	20.38
ZEB1	20.258
TEAD1	18.567
MZF1_1-4	18.257
Stat3	15.876
Myf	15.086
TLX1::NFIC	14.962
EBF1	14.648
INSM1	13.996
HIF1A::ARNT	13.248
Arnt::Ahr	12.928
RUNX1	12.853
NHLH1	12.469
FOXO3	12.339
CTCF	12.192

*z-scores of >10 are considered to indicate highly significant over representation of transcription factor binding sites (TFBS) within the analysed gene list.

Online supplements are not copyedited prior to posting and the author(s) take full responsibility for the accuracy of all data.

E-TABLE 8. CASE ALLOCATIONS FOR SENSITIVITY AND SPECIFICITY OF SVM CLASSIFICATION.

Case	Condition	Sarcoidosis	Tuberculosis	Reactive	Cancer
1	Sarcoidosis	TP	TN	TN	TN
2	Sarcoidosis	TP	TN	TN	TN
3	Sarcoidosis	TP	TN	TN	TN
4	Sarcoidosis	TP	TN	TN	TN
5	Sarcoidosis	TP	TN	TN	TN
6	Sarcoidosis	FN	FP	TN	TN
7	Sarcoidosis	TP	TN	TN	TN
8	Sarcoidosis	TP	TN	TN	TN
9	Sarcoidosis	TP	TN	TN	TN
10	Sarcoidosis	TP	TN	TN	TN
11	Sarcoidosis	TP	TN	TN	TN
12	Sarcoidosis	TP	TN	TN	TN
13	Sarcoidosis	TP	TN	TN	TN
14	Sarcoidosis	TP	TN	TN	TN
15	Sarcoidosis	TP	TN	TN	TN
16	Sarcoidosis	TP	TN	TN	TN
17	Sarcoidosis	TP	TN	TN	TN
18	Sarcoidosis	FN	TN	FP	TN
19	Sarcoidosis	FN	TN	FP	TN
20	Tuberculosis	TN	TP	TN	TN
21	Tuberculosis	FP	FN	TN	TN
22	Tuberculosis	TN	TP	TN	TN
23	Tuberculosis	FP	FN	TN	TN
24	Tuberculosis	TN	TP	TN	TN
25	Tuberculosis	TN	FN	TN	FP
26	Tuberculosis	TN	TP	TN	TN
27	Tuberculosis	TN	TP	TN	TN
28	Tuberculosis	TN	TP	TN	TN
29	Reactive	TN	TN	TP	TN
30	Reactive	TN	TN	TP	TN
31	Reactive	TN	TN	FN	FP
32	Reactive	TN	TN	TP	TN
33	Reactive	TN	TN	TP	TN
34	Reactive	TN	TN	TP	TN
35	Reactive	TN	TN	TP	TN
36	Reactive	TN	TN	TP	TN
37	Reactive	TN	TN	TP	TN
38	Reactive	TN	TN	FN	FP
39	Cancer	TN	TN	TN	TP
40	Cancer	TN	TN	TN	TP
41	Cancer	TN	TN	TN	TP
42	Cancer	TN	TN	TN	TP
43	Cancer	TN	TN	TN	TP

Online supplements are not copyedited prior to posting and the author(s) take full responsibility for the accuracy of all data.

44	Cancer	TN	TN	TN	TP
45	Cancer	TN	TN	TN	TP
46	Cancer	TN	TN	TN	TP
47	Cancer	TN	TN	TN	TP
48	Cancer	TN	TN	FP	FN
49	Cancer	TN	TN	TN	TP
50	Cancer	TN	TN	TN	TP
51	Cancer	TN	TN	TN	TP
52	Cancer	TN	TN	TN	TP
53	Cancer	TN	TN	TN	TP
54	Cancer	TN	TN	TN	TP
55	Cancer	TN	TN	TN	TP
56	Cancer	TN	TN	FP	FN
57	Cancer	TN	TN	TN	TP
58	Cancer	TN	TN	TN	TP
59	Cancer	TN	TN	TN	TP
60	Cancer	TN	TN	TN	TP
61	Cancer	TN	TN	TN	TP
62	Cancer	TN	TN	TN	TP
63	Cancer	TN	TN	TN	TP
64	Cancer	TN	TN	TN	TP
65	Cancer	TN	TN	TN	TP

SVM=Support vector machine, TP=True positive, TN=True negative, FP=False positive, FN=False negative.

Online supplements are not copyedited prior to posting and the author(s) take full responsibility for the accuracy of all data.

E-TABLE 9. CONFUSION MATRICES FOR SENSITIVITY AND SPECIFICITY OF SVM CLASSIFICATION.

Sarcoidosis

	Positive	Negative
True	17	43
False	2	3
Sensitivity (%)	85.0	
Specificity (%)	95.6	

Tuberculosis

	Positive	Negative
True	6	55
False	1	3
Sensitivity (%)	66.7	
Specificity (%)	98.2	

Reactive

	Positive	Negative
True	8	51
False	4	2
Sensitivity (%)	80.0	
Specificity (%)	92.7	

Cancer

	Positive	Negative
True	25	35
False	3	2
Sensitivity (%)	92.6	
Specificity (%)	92.1	

Online supplements are not copyedited prior to posting and the author(s) take full responsibility for the accuracy of all data.

E-TABLE 10. MOST DISCRIMINATING GENES IN SVM CLASSIFICATION.

Top 50 genes for GvNG (SVM1)	Top 50 genes for SvTB (SVM2)	Top 155 genes for CvR (SVM3)
HBG1	IL1RN	ITGB2
PMEL	TMEM114	CHIT1
PDE4DIP	INHBA	TSPAN6
CTNND2	HCAR3	CLDN4
MARCO	OTOA	CTSK
SFN	ACSM5	TPSAB1
MSMB	ELMOD3	TPSD1
PRB4	TREM1	CD5L
MT1H	IL1B	XLOC_002130
CR2	C21orf58	ELF3
TMEM190	CHI3L1	IGFBP2
PITX1	RSAD2	TACSTD2
INHBA	IFIT3	FXYD3
NCR3	ANGPTL4	PERP
FAM26F	ADM	CGN
IGJ	TM7SF4	EPCAM
ZG16B	FOS	CLDN3
IL32	NAMPT	TUSC3
TNFRSF12A	SULF1	TMC4
ENST00000390252	PTGDS	C19orf33
CYP2F1	HES4	SCARNA9
CLDN4	SIGLEC15	DMKN
HTRA4	DUSP1	MLF1
MZB1	DCT	PTPRF
LIPA	CLEC4M	INHBB
MRC1	RASD1	CYR61
MS4A1	CTSK	MMP12
FGD5	CH25H	SPEN
FCRL5	CXCL1	MAL2
DNASE2B	HOXA10	FOLR1
IGLL1	IFI27	KRT19
CTSD	EGR1	LRRC2
PDE6G	LOC401847	TMEM98
FCRLA	CHIT1	MUC1
FBP1	ALOX15B	CRNDE
TK1	CXCL2	F3
CLU	OSM	RHPN2
C4orf7	IFI44L	GPR179
TUBB3	HRASLS2	SCARNA17

Online supplements are not copyedited prior to posting and the author(s) take full responsibility for the accuracy of all data.

FCRL3	SNRNP27	TMEM125
CENPF	IER3	EXPH5
RHPN1	LOC96610	KRT19P2
KRT18	IGLL1	XLOC_011950
HES6	MBD1	EPB41L4B
CTSB	LOC389602	KIAA1522
LCK	CCL4	SIX1
RARRES1	C16orf72	CCDC64B
ELF3	LOC220906	SDC4
C5orf20	C6orf146	CMTM4
LOC96610	CCL21	RIPK4
		C6orf132
		DHCR24
		B3GNT5
		CTSG
		BAIAP2L1
		FOXA1
		IRX5
		CLDN7
		PTPRU
		IGJ
		ZDHHC11
		CEACAM6
		SAA2
		C9orf125
		SAA1
		PRINS
		IRX3
		GPR56
		GPRC5C
		OVOL2
		KCNK1
		CTNNAL1
		PITX1
		PRR15L
		TUBB8
		HDC
		RPA4
		LOC283663
		KRT18
		MGST1
		MAGIX

Online supplements are not copyedited prior to posting and the author(s) take full responsibility for the accuracy of all data.

	RAB25
	PON3
	GOLM1
	PPAP2C
	TEAD1
	TUBB2A
	WDR90
	EHF
	DDAH1
	MPZL2
	EPS8L1
	MARVELD2
	DCBLD2
	ATP9A
	MET
	SH3D19
	PKP3
	KRT18P55
	TRIP13
	FUT3
	DSP
	KIAA0284
	SNX7
	AGSK1
	DDR1
	MYO5C
	FA2H
	S100A14
	FOXQ1
	ID1
	TMEM30B
	PLS1
	STEAP3
	TACC2
	ATP8B1
	B3GNT3
	RDH10
	PARD3
	RAB17
	LIMCH1
	AP1M2
	KCTD1

Online supplements are not copyedited prior to posting and the author(s) take full responsibility for the accuracy of all data.

	ERBB2
	CKMT1A
	XLOC_012568
	SPIRE2
	CD24
	PPP1R16A
	DMRTA2
	NGEF
	FKBP9
	RASEF
	CCL21
	CTSL1
	ANXA8L2
	LOC388152
	SFTA3
	CKB
	PRSS8
	KRT8P12
	TNFRSF21
	KLF5
	CYB561
	CP
	CFB
	MYO1B
	RND3
	PVRL2
	KREMEN1
	PFN2
	EMP2
	DEFB1
	KRT79
	CLC

G=Granulomatous, NG=Non-granulomatous, S=Sarcoidosis, TB=Tuberculosis, C=Cancer, R=Reactive, SVM=Support Vector Machine.

Online supplements are not copyedited prior to posting and the author(s) take full responsibility for the accuracy of all data.
